# A global sampler of single particle tracking solutions for single molecule microscopy

**DOI:** 10.1371/journal.pone.0221865

**Published:** 2019-10-28

**Authors:** Michael Hirsch, Richard Wareham, Ji W. Yoon, Daniel J. Rolfe, Laura C. Zanetti-Domingues, Michael P. Hobson, Peter J. Parker, Marisa L. Martin-Fernandez, Sumeetpal S. Singh

**Affiliations:** 1 Central Laser Facility, Science and Technologies Facilities Council, UK Research and Innovation, Didcot, Oxfordshire, United Kingdom; 2 Department of Engineering, University of Cambridge, Cambridge, United Kingdom; 3 Center for Information Security Technology, Korea University, Seoul, South Korea; 4 Department of Physics, University of Cambridge, Cambridge, United Kingdom; 5 School of Cancer and Pharmaceutical Sciences, King’s College London, London, United Kingdom; 6 Protein Phosphorylation Laboratory, The Francis Crick Institute, London, United Kingdom; Virginia Commonwealth University, UNITED STATES

## Abstract

The dependence on model-fitting to evaluate particle trajectories makes it difficult for single particle tracking (SPT) to resolve the heterogeneous molecular motions typical of cells. We present here a global spatiotemporal sampler for SPT solutions using a Metropolis-Hastings algorithm. The sampler does not find just the most likely solution but also assesses its likelihood and presents alternative solutions. This enables the estimation of the tracking error. Furthermore the algorithm samples the parameters that govern the tracking process and therefore does not require any tweaking by the user. We demonstrate the algorithm on synthetic and single molecule data sets. Metrics for the comparison of SPT are generalised to be applied to a SPT sampler. We illustrate using the example of the diffusion coefficient how the distribution of the tracking solutions can be propagated into a distribution of derived quantities. We also discuss the major challenges that are posed by the realisation of a SPT sampler.

## 1 Introduction

Single molecule imaging is increasingly facilitating high-resolution investigations of molecular motion at the plasma membrane of cells (e.g. [1–4]). Hidden in these data there is crucial information on local environments, transport mechanisms, and the dynamic interactions that regulate protein networks and cell homeostasis. The analysis of particle motion is currently based on fitting trajectories with competing mathematical models, most commonly based on particle mean square displacements (MSD), whose deviations from the linearity characteristic of pure diffusion are interpreted in terms of standard types of particle motion like confined or directed (e.g. [5–9]), but also on hidden Markov calculations [10]. However, the heterogeneity often showed by the trajectories is not easily resolved by model fitting. For the latter to be effective the particles must either maintain the same type motion for multiple consecutive frames (typically >50 frames [8]) and/or display sufficiently long tracks, [5, 10]. This limits SPT to stationary-like conditions or to labelling with quantum dots or fluorescent beads that do not photobleach.

To evaluate particle motion in general one must measure the instantaneous values of motion parameters as they fluctuate along the particle trajectory and ultimately requires single frame sensitivity. The most accurate way to achieve this is from the globally optimal spatiotemporal solution to SPT. In this idealised approach each possible choice of particle reconnections and associated motion parameter values is considered and their consequences compared along the entire length of the tracks, therefore automatically exploiting all the information in the data to output the most likely empirical estimate of reconnections and parameters, and places confidence limits on them. Achieving the globally optimal solution has been the goal of SPT for decades but it has proven to be computationally prohibitive because of the colossal size of the configuration space of particle reconnection possibilities at high particle density, low signal-to-noise ratio (SNR) and fast particle movement typical of single molecule images in cells [11]. A wide range of methods has been developed to address this problem [12]. Naïvely one may deduce that it roughly scales as the factorial of the number of particles (thousands), motion parameters (dozens), and frames (hundreds). To make the problem tractable previous algorithms reduced the size of the configuration space both by imposing a priori narrow bounds on the parameters, from modelling or previous knowledge, and by approaching the globally optimal solution by taking many locally optimal solutions (e.g. [13–16]). This typically produces ‘tracklets’ separated by gaps, after which longer tracks may be recovered, for example, via minimal path techniques (e.g. [17, 18]), or maximum likelihood methods (e.g. [3, 4, 19, 20]). Although these algorithms addressed many of the challenges from high particle density and low signal-to-noise, it is difficult to ascertain how sensitive the results are to their choice of parameters [11], and the loss of temporal globality hinders access to the very statistical information one requires to evaluate dynamic motion.

Here we present the Biggles tracker, an automatic Bayesian Inference-based, Gibbs-sampler, GLobal EStimator of particle tracks and parameters that converges towards the globally optimal spatiotemporal solution in a computationally time practical for real-world tracking. Biggles allows to estimate the uncertainty in the tracking solution and finds probable alternative solutions. It therefore opens the possibility to propagate the tracking error to the estimation of derived biophysical quantities such as diffusion coefficients.

## 2 Material and methods

Biggles uses some data, *Y*, which are the spatial and temporal (*x*, *y*, *t*) coordinates of the single particle spots detected in the images (referred to as *observations*), a hypothesis for the assignment of these observations to tracks, the track partition *ω*, and some global parametrised model, *θ*, for the properties of the system. (Full details of the algorithm are in Supporting information S1 Appendix). We write *N* for the number of observations, *T* for the number of time points, which in many applications is the number of images taken, and Ω for the set of all valid track partitions. We can fold into the algorithm data for any imaging detector and allow for a set of spurious measurements in each frame. We use a flexible yet simple model for particle motion: a random-walk [21]. Observations that are deemed to be spurious are collectively referred to as clutter. The clutter is treated as part of the track partition in the sense that in any partition each observation is assigned either to exactly one track or to the clutter. A track is defined in terms of the observations assigned to it, which must number at least two, and in terms of its first and last time points, which need not be associated with observations. At any time point a track may have at most one observation. A pair of consecutive observations within a track, which need not occur at consecutive time points, is referred to as a *link*. Therefore each track has at least one link.

Biggles finds the probability of any given set of tracks and motion parameters given the data. In a Bayesian framework [22], this allows one to explore via sampling the joint track and parameters empirical (or posterior) probability function:

$$\begin{array}{c} P(\omega ,\theta |Y), \end{array}$$ (1)

with the aim of identifying the most probable set of tracks and parameters, together with the uncertainties on them. We note that, given that tracks are defined by observation and first and last time points, two different sets of tracks may correspond to the same partitioning of the observations. Although Biggles formally samples tracks, we will usually ignore this distinction in the subsequent discussion and denote them simply by the (track) partition *ω*. To avoid having to sample from the posterior simultaneously Biggles uses a Gibbs sampler [23], which allows one to draw samples *ω*_*i*_ and *θ*_*i*_ alternately from two conditional distributions:

$$\begin{array}{ccc} \omega_{i} & ∼ & P(\omega |\theta_{i-1},Y), \end{array}$$ (2)

$$\begin{array}{ccc} \theta_{i} & ∼ & P(\theta |\omega_{i},Y). \end{array}$$ (3)

**Fig 1:**
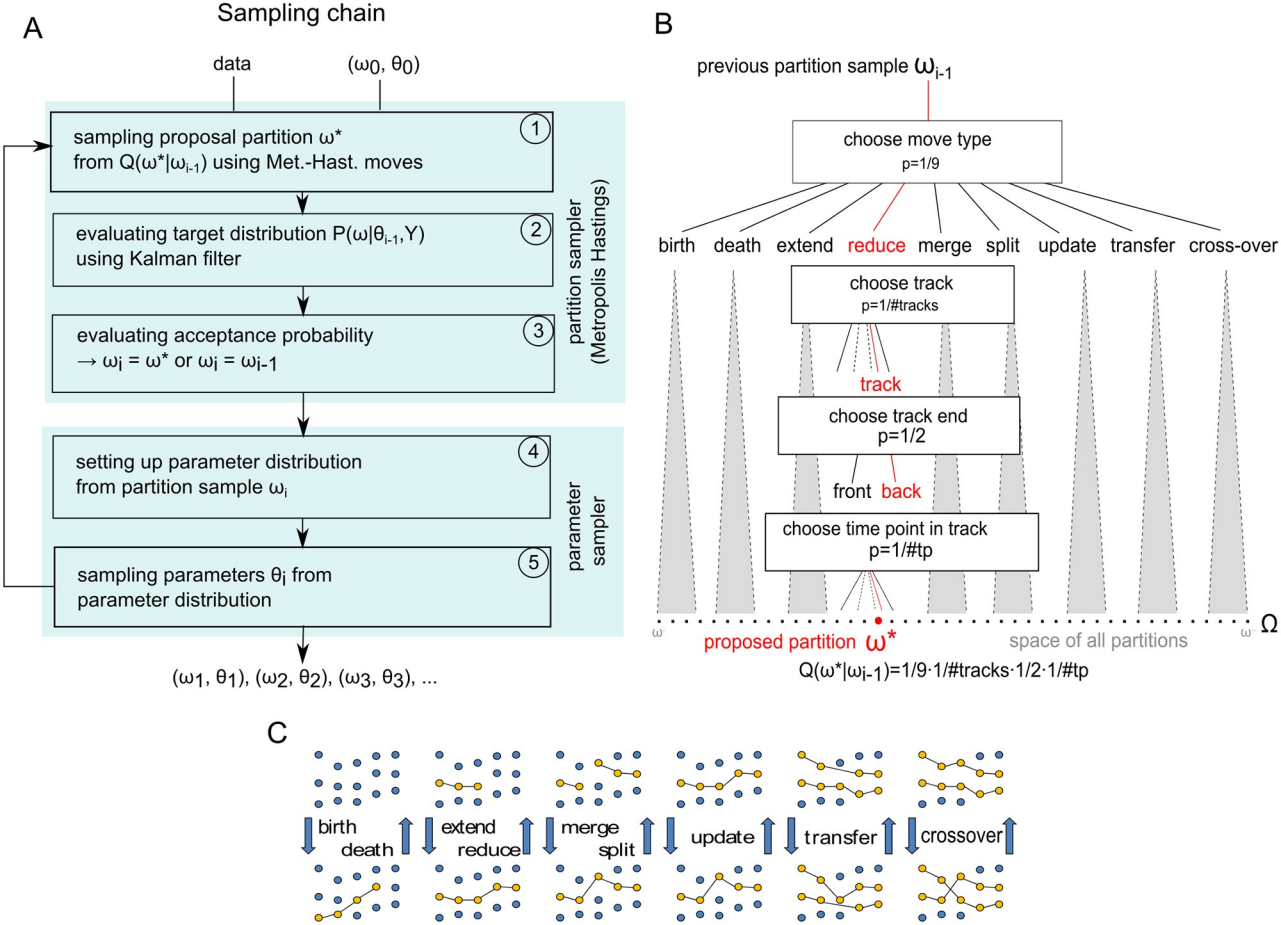
Algorithm overview. A) Biggles runs two sampling chains with different initial partitions *ω*_0_. Each chain is a Gibbs sampler alternately sampling partitions, steps ①-③, and parameters, steps ④-⑤. B) Schematic of sampling and evaluating the proposal distribution *Q*(ω*|*ω*_*i*−1_), step ① in panel A. The sampling is realised by descending a sampling tree; the root of the tree is the current partition *ω*_*i*−1_ and the leaves are the partitions ω* that can be reached, i.e. where *Q*(ω*|*ω*_*i*−1_) > 0. Each branch has a certain probability given the parent branch, so that the probability of a leaf is the product of the probabilities of the branches traversed during the descent. A descent down the reduce move branch is sketched. Other move types are executed in a similar manner, but with different branching operations. C) Cartoon of the move type pairs. Each move type has a positive probability to undo any modification of its partner, i.e. *Q*(ω*|*ω*_*i*−1_) > 0 if and only if *Q*(*ω*_*i*−1_|ω*) > 0. D) Cartoon of the observation likelihood calculation in step ② in panel A, using the update move example of panel C. The Kalman filter estimates the particle states (black dots) of the track model; the red line illustrates a possible course. The likelihood of the observations assigned to the track (yellow) is calculated using the filter’s observation model. The change in the track assignment by the update move leads to different state estimates and hence to different observation likelihoods. For full details see Supplementary Notes.

An overview is shown in Fig 1A. We note that the partition sampler (steps ①-③ in Fig 1A) draws samples from a *probability mass function* (PMF), while the parameter sampler (steps ④ & ⑤ in Fig 1A) draws samples from a *probability density function*. The parameter space *θ* has 7 dimensions, while Ω has no intrinsic dimensionality, but is of finite size. While the parameters *θ* can be sampled directly because their posterior distribution is known and separable, sampling from the track partition is non-trivial, as only a small number of analytical PMFs have known direct sampling algorithms. We therefore use the Metropolis-Hastings algorithm [24], which can draw samples from almost any PMF, and which, on convergence, yields candidate-sets of tracks whose distribution matches the track partition posterior *P*(*ω*|*θ*, *Y*).

The *acceptance probability* of the Metropolis-Hastings sampler (Fig 1A step ③) is given by

$$\begin{array}{c} \min(\frac{P(\omega^{*}|\theta_{i-1},Y)}{P(\omega_{i-1}|\theta_{i-1},Y)}\frac{Q(\omega_{i-1}|\omega^{*})}{Q(\omega^{*}|\omega_{i-1})},1), \end{array}$$ (4)

where the proposed new track partition ω* is sampled from the *proposal mass function* *Q*(ω*|*ω*), Fig 1A step ①. The sampling uses a step-by-step approach which can be described as descending a tree, Fig 1B. The root of the tree is the last partition sample *ω*_*i*−1_. The nine move types are the main branches of which one is randomly chosen. Cartoons of the move types are shown in Fig 1C. The further steps depend on the sampled move type. For example, in the execution of a “reduce” move, the track to be shortened is chosen, then the end of the track is sampled (front or back) and finally the time point within the track where the cut happens is sampled, Fig 1B. Each step has a probability that depends on the previous step. The probability to sample the proposal partition is the product of the probability of each step. In the example *Q*(ω*|*ω*_*i*−1_) = 1/9×1/(number of tracks)×1/2×1/(number of time points in the selected track where the cut is allowed). The sampling structure ensures that

$$\begin{array}{c} \sum_{\omega \in \Omega }Q\left(\omega |\omega^{\prime }\right)=1\;\text{for}\;\text{any}\;\omega^{\prime }\in \Omega , \end{array}$$ (5)

which is a necessary condition for *Q* being a PMF. As already mentioned above, for a valid track we require that it contains observations at a minimum of two time points (moves that create tracks with fewer than two observations are not allowed), and we note that two tracks having the same observations and therefore the same links may not be equal, since a track may have unobserved states before the first or after the last observation. To improve the performance of the algorithm, we also limit the maximum speed of the particle (to a value that is larger than any value that can be physiologically expected).

It is possible that the execution of a move does not lead to a valid track partition. For instance, at the end of the birth move, the created track may contain no observations purely by chance. In such a case ω* is set to *ω*_*i*−1_. Since in such a case accepting and rejecting leads to the same result, *ω*_*i*_ = *ω*_*i*−1_, we regard the proposal as *identity* as opposed to *accepted* or *rejected*. It means that *Q*(*ω*|*ω*) > 0 for most *ω*. We tried to minimise the identity proposals in the move design, e.g. we do not attempt a death move if the partition contains no tracks or we do not attempt to split a track that has only three observations and so on. The occurrence of the identity proposal is not a theoretical novelty. *Q*(*ω*|*ω*) > 0 also occurs if *Q* is a Gaussian distribution. However since the Gaussian distribution is continuous rather than discrete, it is very unlikely that a proposal sampled from it is equal to the last sample and special considerations of such a case are unnecessary. The distinction only takes effect in the calculation of the acceptance rate, where identity proposals are treated as rejected proposals even though their acceptance probability is equal to one.

To evaluate the target distribution, *P*(*ω*|*θ*_*i*−1_, *Y*), of the Metropolis-Hastings sampler, Fig 1A step ②, we expand it into three different components using Bayes’ theorem,

$$\begin{array}{c} P(\omega |\theta ,Y)\propto P(Y|\omega ,\theta )P(\omega |\theta )P(\theta ), \end{array}$$ (6)

where *P*(*θ*) are the parameter priors and *P*(*ω*|*θ*) is the probability of the track partition, which takes the assignment of observations to tracks into account but not their physical properties. *P*(*ω*|*θ*) is assumed to depend separably on four parameters in *θ*, according to closed-form distributions. The likelihood *P*(*Y*|*ω*, *θ*) is factorised into the likelihood of the clutter observations *P*(*Y*^0^|*k*_0_, *θ*) and the product of the likelihoods of the observations assigned to tracks, $\prod_{i=1}^{K}P\left(Y^{i}|k_{i},\theta \right)$, where *K* is the number of tracks and *Y*^*i*^ are the observations assigned to track *k*_*i*_. The likelihood *P*(*Y*^*i*^|*k*_*i*_, *θ*) is evaluated using a state space approach [25]. The (unobserved) particle state encompasses position and velocity, $X={\left(x,\dot{x},y,\dot{y}\right)}^{T}$. The particle motion is a modelled as random walk in the positions plus a velocity term, where the velocity follows its own random walk. This allows for both directed motion and undirected motion, but also more complicated types of motion. We use the Kalman filter [26] to estimate the states of the particles, both at time points where the track was observed and at time points without observations, e.g. due to fluorophore blinking. To base our estimates on all observations assigned to the track, we apply the Rauch-Tung-Striebel backwards smoothing filter [27]. The observation likelihood is calculated by three principle steps:

1. Assigning the observations to tracks (done by the partition sampler),
2. Estimating the track’s states from the observations of the track using the Kalman filter (i.e. inferring the model),
3. Calculating the likelihood of the observations given the model (i.e. states and the error estimates).

**Fig 2:**
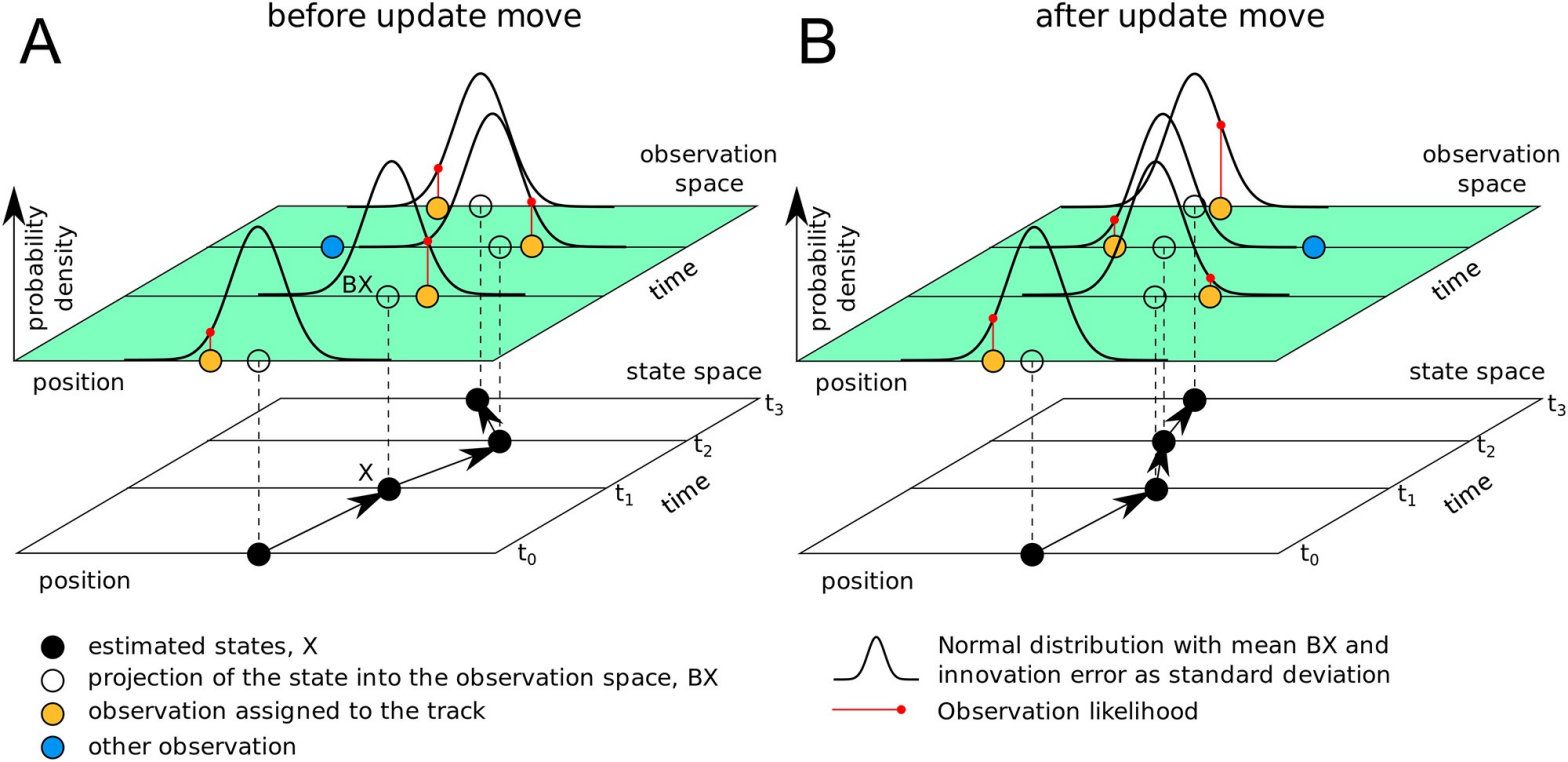
Principle of the observation likelihood calculation. The partition sampler has assigned the observations (yellow) to a track. From those observations, the Kalman filter estimates the most likely states (black) and the state estimation errors (not shown) given the model of particle motion. Under this fitted model, each observation assigned to the tracks has a specific likelihood, which is evaluated from a normal distribution that is centred in the projection of the state into the observation space and whose standard deviation is a combination of the observation error and the state estimation error. When the partition sampler reassigns an observation at time *t*_2_ of the track by executing an update move, the fresh application of the Kalman filter results in new estimates for all states. That leads in turn to different observation likelihoods even for those observations whose assignment has not changed.

An example is depicted in Fig 2. The figure highlights the change in the observation likelihood caused by an update move. The sample from the proposal distribution contains the track given by four observations in Fig 2A. A fifth observation at time point *t*_2_ is considered clutter. The Kalman filter estimates the particle states from the observations of the track, which are marked by black dots in the state space. The states and their error estimates in turn imply how likely it is to find the observations at their actual positions. The likelihood of the individual observation $Y_{j}^{i}$ is given by a normal distribution $N(Y_{j}^{i};B\hat{X_{j}^{i}},\hat{S_{j}^{i}})$, where $B\hat{X_{j}^{i}}$ is the projection of the state $X_{j}^{i}$ that is associated with $Y_{j}^{i}$ into the observation space and $\hat{S_{j}^{i}}$ is the innovation error, which is composed of the observation error *R* and the state estimation error. The full equations are given in the supplied appendix 5 sections 3.2 and 3.3. In Fig 2B, the partition sampler has swapped the two observations at time *t*_2_; the observation that previously was clutter is now part of the track and the other observation is now considered clutter. The Kalman filter provides new state estimates of all observations of the track, which leads in turn to new observation likelihood estimates. For example the likelihood of the observation at *t*_1_ is reduced after the update move, while the likelihood of the observation at *t*_3_ is increased. An example showing observations and estimated states of a track is giving in S7 Fig.

The 2 × 2 covariance matrix of the observation noise *R* that contributes to the innovation covariance is sampled as part of the parameter sampling stage of the Gibbs sampler, Fig 1A step ⑤. The details about the calculation of *P*(*ω*|*θ*_*i*−1_, *Y*) are described in the supplementary notes.

In addition to the three parameters that determine *R*, the further four parameters in *θ* control the track partitioning. The birth rate λ_*b*_ is the average number of tracks that begin at time point *t* in a normalised area *A*. The clutter rate λ_*c*_ is the average number of spurious observations that are found at time *t* in a normalised area *A*. We use the symbol “E” for the unit of the rates and set 1E equal to 1 event per frame and 100pixels×100pixels. The observation probability *p*_*o*_ is the probability that a particle is observed at time *t* and the survival probability *p*_*s*_ is the probability that a track that is present at time *t* − 1 is also present at time *t*. We note this implies the assumption that the track length is exponentially distributed with mean 1/(1 − *p*_*s*_). This is not a limitation for imaging fluorophores, since it reflects the bleaching behaviour of fluorescent molecules. Quantum dots, which are used in bioimaging as well, do have for practical purposes an infinite life time. The posterior distribution *P*(*θ*|*ω*_*i*_, *Y*) can be sampled directly and is separable, Fig 1A step ④-⑤. In particular, the rates λ_*b*_, λ_*c*_ and the probabilities, *p*_*o*_, *p*_*s*_ are assumed to follow Gamma and Beta distributions, respectively, where the parameters for the Gamma and Beta distributions are derived from the current partition sample by counting the number of tracks, their lengths and so on, Fig 1A step ④. The observation noise of the Kalman filter, *R*, is sampled from an inverse Wishart distribution, where the parameters are derived from the track observations and the track model. In summary we have

$$\begin{array}{c} \theta =(\lambda_{b},\lambda_{c},p_{o},p_{s},R). \end{array}$$ (7)

The initial samples *θ*_0_ are initialised to some values. In principle we need only specify loose “plausible” bounds of *θ*_0_. Our current implementation initialises *θ*_0_ to uncontroversial typical values. The rates λ_*b*_ and λ_*c*_ have improper uninformative priors on them being positive, *p*_*s*_ and *p*_*o*_ have uniform priors over (0, 1) and *R* has an inverse Wishart prior W(Φ,s), see also Supplementary Notes. Following the parameter sampling, the cycle is complete and a new track partition is sampled from the proposal distribution.

**Fig 3:**
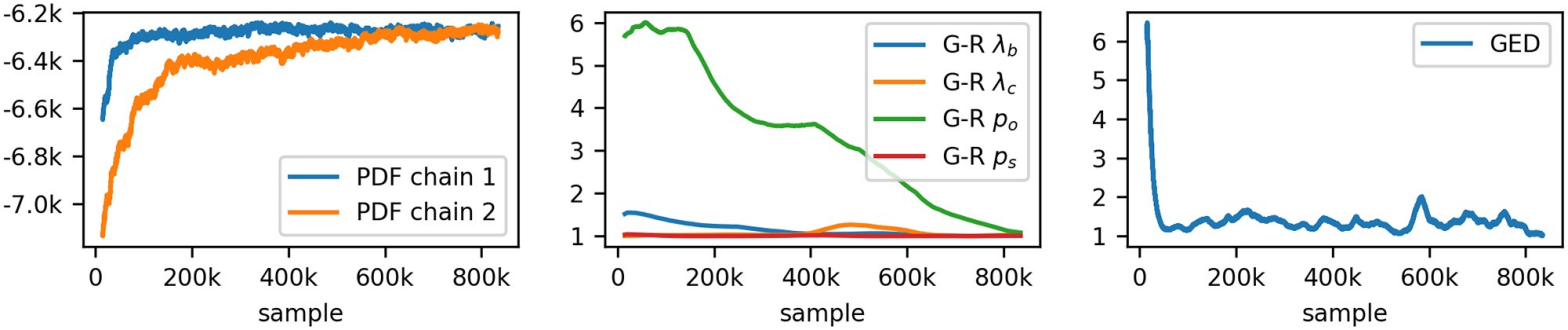
Convergence. The panels show: the posterior density of the two chains; the Gelman-Rubin statistics for the four tracking control parameters and the GED criteria, (average-cross-chain-GED)/(sum-of-average-inner-chain-GEDs).

A Biggles chain is ergodic, which is shown in the supplementary information. The initial part of the sampling before the sampling chain has reached the limiting distribution (the target distribution) is called burn-in phase. In order to assess the convergence to the limiting distribution of the Metropolis-Hastings sampler we run two chains. The first chain starts with the partition without any links, i.e. where all observations are assigned to the clutter. This is the *minimum partition, ω*^0^. For the second chain, we use a randomised greedy algorithm to assign as many observations as possible to tracks to create the initial partition. No further links can be added to such a partition, which is therefore referred to as a *maximum partition*. Usually, Ω has many maximum partitions. The sole purpose of this approach is to get two starting partitions that are far away from each other. To assess the convergence of the two chains we implemented two tests. The first test is based on the similarity of partitions. We consider a track partition as a graph, where the observations are the nodes and the links are the edges. We use the graph edit distance (GED) [28] as similarity measure, where link insertion and link deletion are the graph edit operations. In other words, we define distance between two track partitions as the number of links in which the two partitions differ. For convergence we demand that the average cross-chain GED does not exceed the sum of the averages of the two inner chain GEDs. Second, the Gelman-Rubin [29, 30] statistics is implemented for the parameters λ_*b*_, λ_*c*_, *p*_*o*_ and *p*_*s*_. Fig 3 shows example data for the convergence.

By design the Metropolis-Hastings partition sampler will yield correlated samples. That reduces the effective sample size [30] and increases the total number of samples required. On the other hand the number of samples that can be recorded is constrained by the computational resources. Specifically, partition samples can be dozens or hundreds *kB* large, depending on the number of observations, *N*. Therefore we record 1 in every *n* samples, thereby *thinning* the chain, where *n* linearly depends on the acceptance rate at the end of the burn-in phase. In the present implementation every 1 in 8 samples would be recorded if the acceptance rate were 25%.

The burn-in phase is longer than the target distribution sampling phase. Experience shows that the ratio of the number of target distribution samples to the number of burn-in samples has a median of about 3.5% and a mean of 5.5%. The maximal observed value is 25%.

At the point of convergence, the Gibbs sampler starts to sample candidate-sets of tracks and parameters whose distribution matches that of the joint posterior distribution. We may therefore interpret results such as the sample mode and sample variance as maximum empirical estimates and experimental errors respectively. The ability of Biggles to directly return a representative sample of tracking results and parameters, and thus to place confidence limits on these, is powerful and, as far as we are aware, it is novel in this field.

To demonstrate Biggles we use synthetic and single molecule microscopy (SMM) data sets. For our simulations, we first generate tracks with a given birth rate $\lambda_{b}^{*}$ and survival probability $p_{s}^{*}$, and the states of the particles at each time point of the tracks is determined by the state dynamics. The tracks of states are referred to as ground truth. Next, track observations are created with probability $p_{o}^{*}$ from the states using a Gaussian observation model, $N(0,R^{*})$, and the clutter observations are created, governed by $\lambda_{c}^{*}$. Finally, the data is cropped to the field of view. This final result is the *realisation of the ground truth* (GTR). That means we have three stages of ground truth; the parameters, the tracks states simulated from these parameters and the observations created from the states. We enable a range of behaviours in the simulation; random walk, directed motion, a combination of both and track splitting.

If not mentioned otherwise, the unit of *x* and *y* is pixel, where one pixel is 160nm×160nm for the SMM data sets that we present. The unit of time is the frame index. The time lag between two frames is 0.05 seconds for the SMM data sets.

## 3 Results

**Fig 4:**
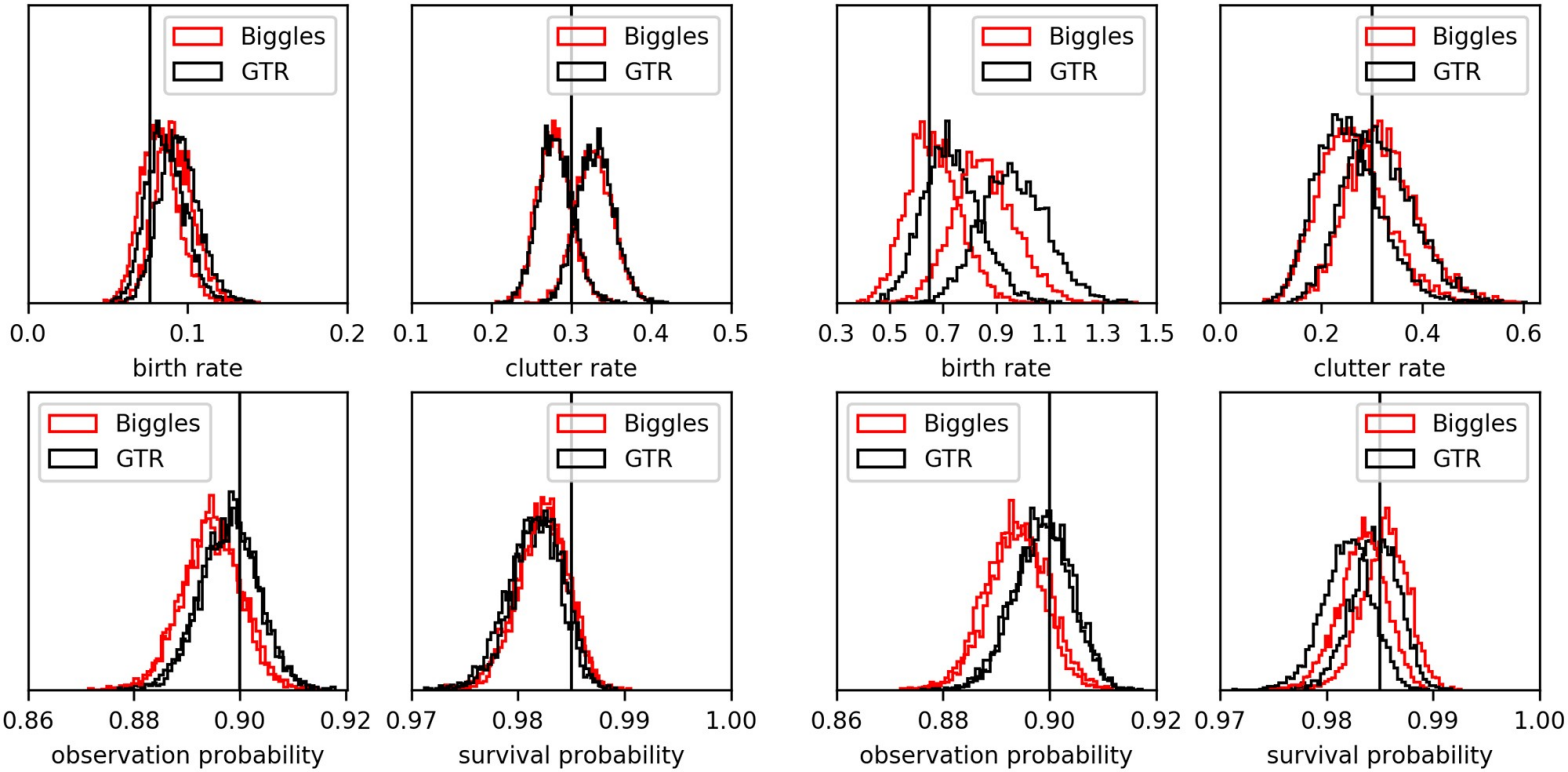
Recovery of the simulation parameters. The histograms of the parameter samples for the GTR (black) and the parameter samples created by Biggles (red). A pair of data sets is shown with low track density (left) and a pair with high track density. The ground truth parameters for each pair are indicated by black vertical lines. See supplementary information for Q-Q plots for series of 10 such data sets.

We created synthetic data sets to test the correctness of the Biggles sampling. We simulated two series of 10 data sets each that differ in the birth rate, with $\lambda_{b}^{*}=0.1E$ and $\lambda_{b}^{*}=1.6E$ respectively. To get a similar observation count for both series we reduced the field of view in the series with the higher birth rate. For each birth rate, Fig 4 shows the posterior distribution of λ_*b*_, λ_*c*_, *p*_*o*_ and *p*_*s*_ for two of these data sets in comparison with parameter samples given the GTR, *P*(*θ*|*GTR*, *Y*). The ground truth value for the parameter is indicated by a vertical line. First we see that the distributions derived from the GTR are are well distributed around the ground truth parameter values, albeit with some bias in the survival probability. Moreover, we observe very good agreement between the Biggles posterior distributions and those derived from the GTR (see also the Q-Q plot in the support material S1 Fig). There are some offsets in the birth rate and the observation probability, and also in the survival probability in the higher crowding case. In other words Biggles has a slight bias to fewer, longer tracks with less observations (i.e. long tracks with many dark states), while the total number of observations in tracks remains equal to that for the GTR. For example, if two short tracks with a temporal gap are merged, then survival rate goes up, the observation rate and the birth rate go down, while the number of observations in tracks remains unaffected.

We calculate the frequency with which any two observations have been linked and use it as probability estimate, $\hat{p}\left(l\right)$, for the occurrence of a link, *l*,

$$\begin{array}{c} \hat{p}\left(l\right)=\left(\text{number}\;\text{of}\;\text{records}\;\text{containing}\;l\right)/\left(\text{total}\;\text{number}\;\text{of}\;\text{records}\right). \end{array}$$ (8)

**Fig 5:**
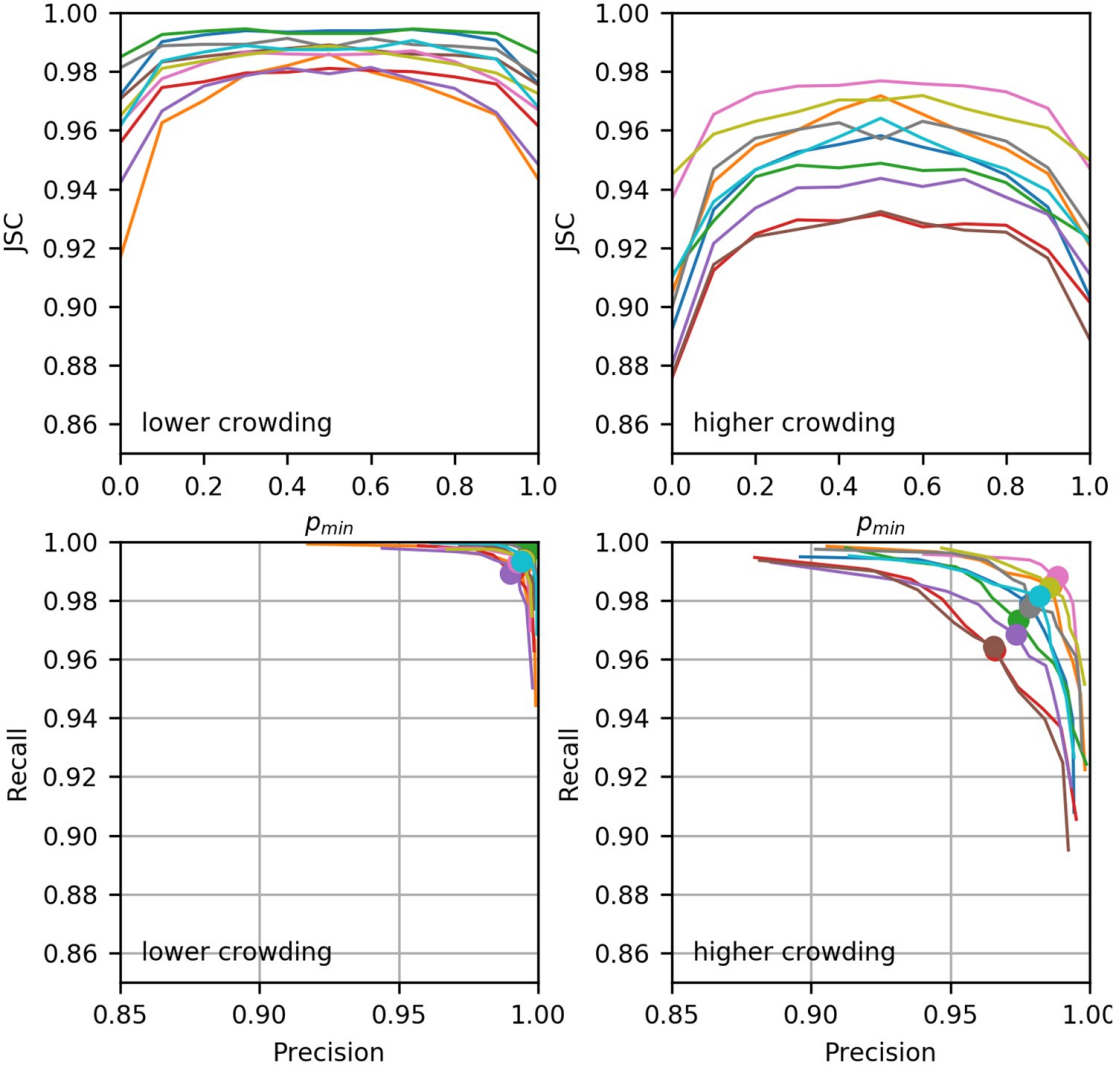
Performance of Biggles tracking for data sets with low and high crowding. From left to right: JSC for less crowded data sets, JSC for more crowded data sets, recall against precision (lower crowding) and recall against precision (higher crowding). The dots in the two right panels mark the values at *p*_*min*_ = 0.5.

To assess the tracking results we adopted performance measure from [31]. However a direct usage of these measure is not possible since there are not designed for tracking PMF. We adopted the Jaccard similarity coefficient (JSC), in the following way. For a given ratio, *p*_*min*_, we consider a link, *l*, as predicted if $p_{min}\leq \hat{p}\left(l\right)$. We express the results in terms of the confusion matrix as true and false positives and negatives; TP, FP, TN and FN. For example, let *p*_*min*_ = 0.6 and let *l* occur in the GTR. If $\hat{p}\left(l\right)=0.7$ then *l* counts towards the number of TP. If $\hat{p}\left(l\right)=0.5$ then *l* counts towards the number FN. However, if *p*_*min*_ = 0.5 and $\hat{p}\left(l\right)=0.5$ then *l* counts as TP. For any *p*_*min*_ we can calculate JSC = TP/(TP + FP + FN), and also recall = TP/(TP + FN) and precision = TP/(TP + FP). All measures have a range between 0 and 1, where 1 is best and 0 is worst. If *p*_*min*_ = 1 then only links that occur in all records are considered positives. The number of FP is lowest and FN is highest. When *p*_*min*_ is reduced, FP will increase and FN will drop. If *p*_*min*_ = 0 then any link that at least occurred in one sample will be considered as positive, which gives a large number of FP, while ideally the number of FN should go to zero. In other words, if a link is in the GTR then we expect it will at least occur in one sample. The two left panels of Fig 5 show the JSC response for the two series of data sets with $\lambda_{b}=0.1E$ and $\lambda_{b}=1.6E$ respectively. We observe that the JSC generally is highest for 0.4 < *p*_*min*_ < 0.6, while it significantly drops for *p*_*min*_ near 0 and 1. For the less crowded data set we find higher JSC (between 0.97 and 0.99 at *p*_*min*_ = 0.5) than for more crowded data sets (between 0.92 and 0.98 at *p*_*min*_ = 0.5). The two right panels of Fig 5 show the precision vs. recall plots. The values at *p*_*min*_ = 0.5 are marked with a dot. We observe that with lower *p*_*min*_ the recall increases, i.e. less GTR links are missed, while with higher *p*_*min*_ the precision increases, i.e. less false predictions are made. For the data sets with lower track density we observe recall and precision higher than 0.99 at *p*_*min*_ = 0.5 and for data sets with higher track density we observe recall and precision values of at least 0.96 at *p*_*min*_ = 0.5. For some low track density data sets we observe almost perfect recall at *p*_*min*_ = 0 and almost perfect precision at *p*_*min*_ = 1. However also for the higher density data sets we get recall and precision of at least 0.99 at the extreme ends of *p*_*min*_. We did not calculate the receiver operating characteristic (ROC) curve, since we did not calculate the number of TN. However, the plot recall versus precision is a similar visualisation; rather than assessing how many negatives have been falsely classified as positive as in ROC, we assess how many of the classified positives are true positives.

**Fig 6:**
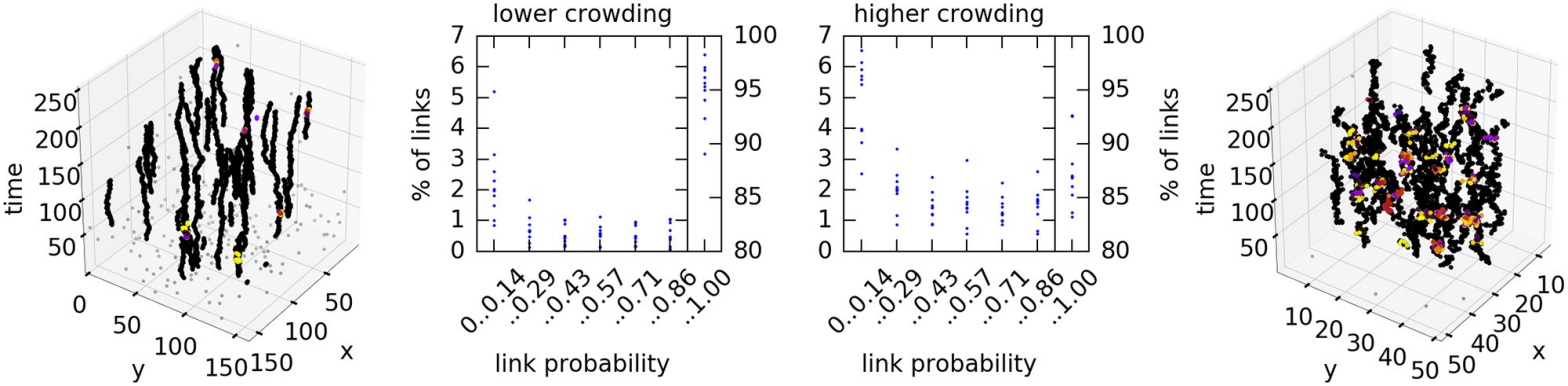
Change of the distribution depending on the track density. The left histogram shows the link probability of 10 data sets with lower birth rate. One of the data sets is shown in the left 3D plot. The histogram on the right shows the link probability of 10 data sets with higher birth rate. In comparison with the low-birth-rate data sets, the high-birth-rate data sets have fewer links with very high probabilities, while the number of links with medium and low probability is increased. This higher uncertainty of some links is due to the higher crowding.

We use the synthetic data of Fig 4 to illustrate the dependency of the posterior distribution on the track density, see Fig 6. The links shown on the left of Fig 6 are from one of ten data sets with low birth rate (3429 observations) and, on the right is one of the data sets with high birth rate (3558 observations). The histograms in the middle panels shows the percentage of links against their estimated probability of occurrence. The low birth rate data sets have a higher proportion of uncontroversial links, with 7 data sets having 95% or more compared to the data sets with high birth rate with 9 data sets having less than 90% of uncontroversial links. We see more links with low probability. The increase in links with medium probability indicates that we observe an increased uncertainty in our estimate of the tracking result.

Single molecules show a variety of modes of motion. While we did not fully explore the behaviour of our algorithm under such conditions, we do provide some illustrative tracking examples in S2–S5 Figs; molecules that change the mode of motion from random walk to direction motion or the other way around (S2 Fig); a mixture of molecules some of which move in a random walk and some of which have a directed motion (S3 Fig); a mixture of molecules that move in a random walks with two different diffusion constants (S4 Fig) and random walks of molecules with different local densities (S5 Fig). All these synthetic data sets have been analysed without special adjustments of the algorithm.

**Fig 7:**
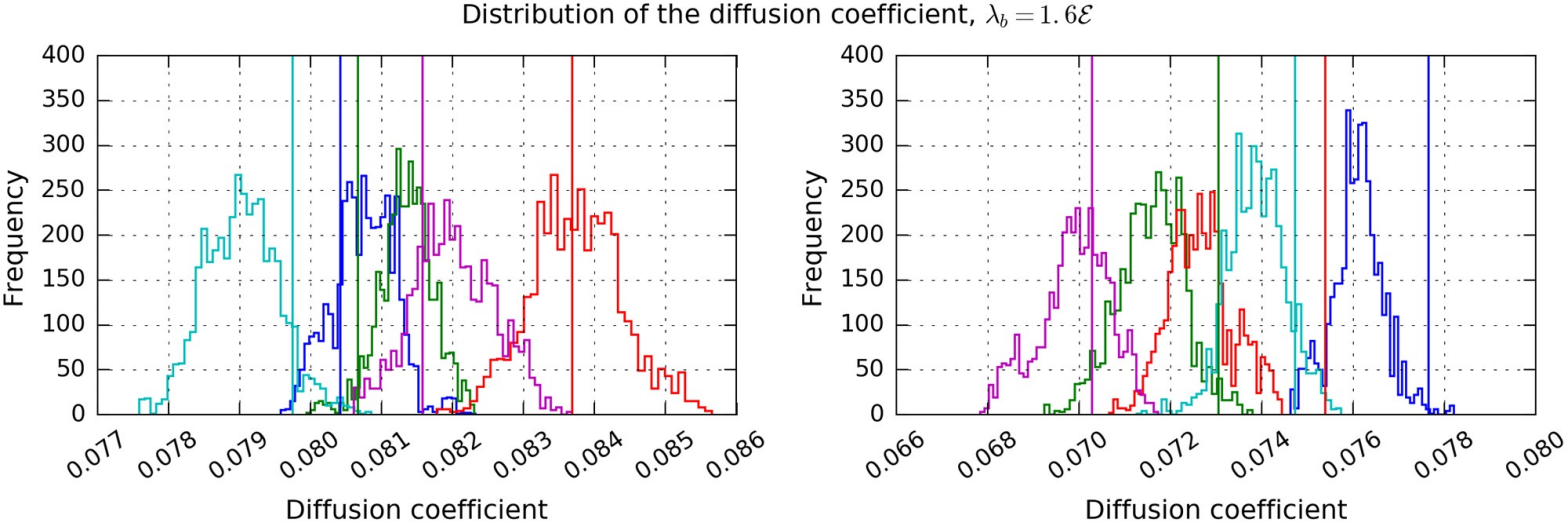
The distribution of diffusion coefficient, *D*, calculated from the Biggles samples. Each panel shows the result from a single GT simulation, i.e simulated particle states governed by birth rate and the survival probability. From each GT ten GTR have been created, governed by the observation rate, the observation error and the clutter rate. The ground truth *D* = 0.08, *D* calculated from the GTR is shown as vertical, dashed lines whose colours correspond to the colour of the histograms. The two numbers indicate how often the GTR *D* lies within 1*σ* and 2*σ* of the sample average of the respective Biggles output. Since there are 10 GTR per panel, possible values are multiples of 10.

We demonstrate the sampling of a derived quantity using the example of the diffusion rate [32], see Fig 7. We created 100 data sets as before with a birth rate of 1.6E. We calculated a single diffusion coefficient, *D*, for the GTR as well as for every recorded sample. The diffusion coefficient was calculated from the mean squared displacement, 〈*r*^2^(*τ*)〉, for time lag *τ*. The estimate is calculated from the positions of the observations assigned to the tracks. The mean was taken over all tracks, see Supplementary Notes. Each of the two panels show the data of five realisations of a ground truth partition (GTR), i.e. 5 sets of observations generated from the same ground truth states. The diffusion coefficient of each GTR is shown by a vertical line. They are different for each realisation due to the random nature of the generation. Each set of observations was used as input for Biggles and *D* was estimated for each recorded sample, 4000 samples per run. The *D* of the samples are shown as histograms in the same colour as the related *D* of the GTR. We calculated to each data set confidence intervals (CI) and counted how often it contains the *D* of the GTR. The CI to confidence level *X*% is calculated as the smallest interval that contains at least *X*% of the samples. We found an agreement of 52% for a 70% CI and 75% for a 95% CI. This is a very encouraging result. However the procedure somewhat underestimates the errors. This will be subject to future investigations.

**Fig 8:**
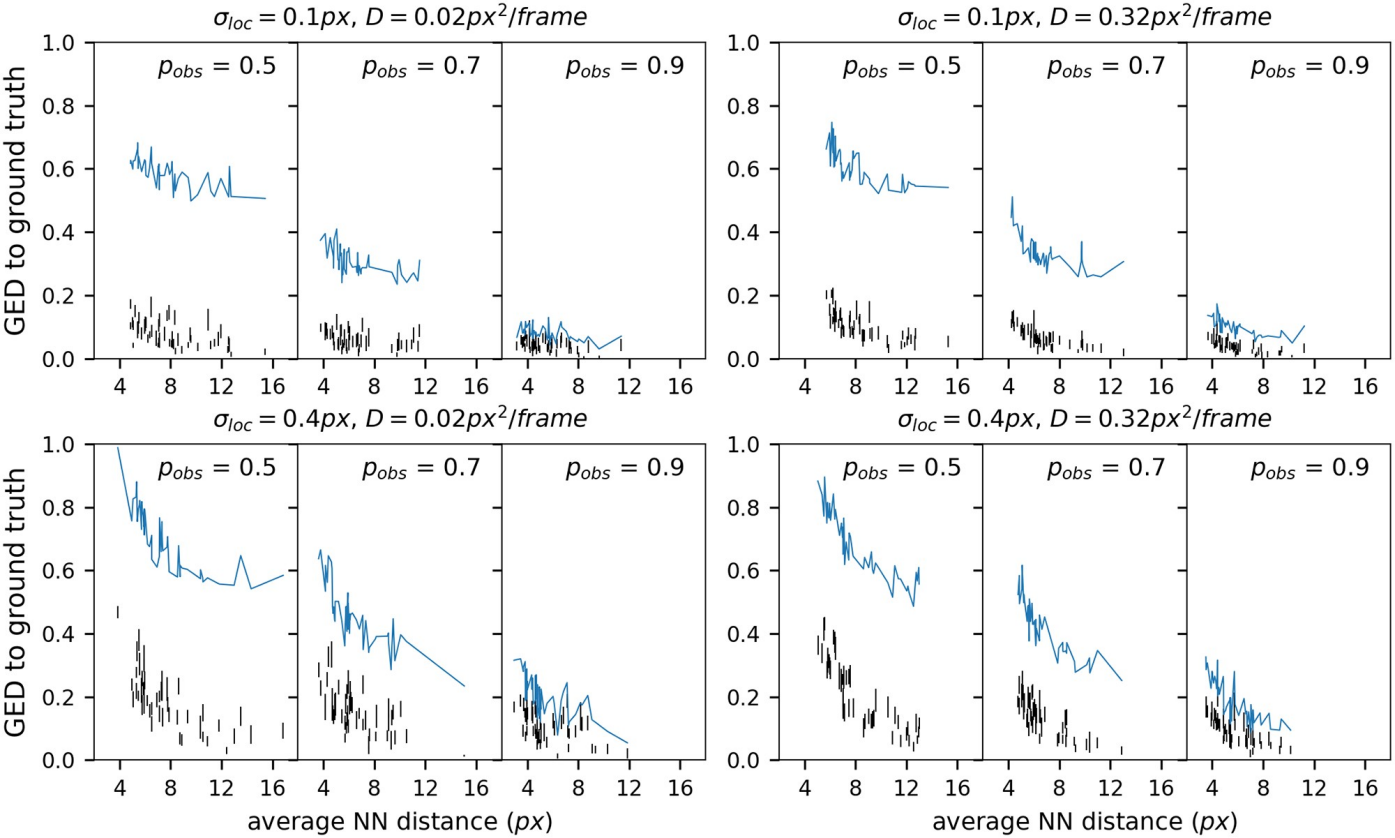
Comparison between Biggles and uTrack. The uTrack results are shown in blue, one point per tracking result, the points connected by a line. The Biggles results are shown as vertical black lines, each line represents a single tracking result connecting the value of best with the value of the worst sample. The GED to the ground truth (number of wrong links + number of missed links) is normalised by the number of observations of the data set.

We compared Biggles with uTrack [20]. For the comparison we simulated particles at a range of different conditions Fig 8. The track density was simulated by assuming different birth rates with spatially uniformly distributed first observations. The average nearest neighbour (NN) distance of the observed particles was determined from the GTR. It varies roughly from 4*px* to 12*px*. Simulated particles moved in a random walk (*D* = 0.02*px*^2^/*frame* and *D* = 0.32*px*^2^/*frame*). After the generation of the GT particle trace the observation model was applied. A particle was observed at any time with a given probability, *p*_*o*_ ∈ {0.9, 0.7, 0.5}. The particle observation was sampled from a normal distribution with the GT particle location as mean. We assumed two different localisation errors, 0.1*px* and 0.4*px*. The life time of the particle tracks was exponentially distributed. We added uniformly distributed spurious detection which resulted in a density of 0.4±0.1 observations per 100*px*×100*px* and frame. The resulting data sets had on average 2003±575 observations.

The results where compared with the GTR. The number of links in which the trackers differed from the GTR, the GED, was normalised by the number of observations in the input data. Note that the value can be larger than 1, with 2 being an upper boundary. A value of 0 indicates total agreement. For uTrack a single value per data set is shown in Fig 8 (blue), for Biggles each tracking result is represented by a vertical black line representing the range of all samples (2000 per data set). As expected, both trackers perform well under good conditions and the performance slides if the condition get worse. For a localisation error of 0.1*px* and high observation probability both trackers perform very well. Biggles remains stable for a low localisation error, even if the observation probability drops. The uTrack tracker on the other hand drops in performance if the observation rate goes down. For a localisation error of 0.4*px* the tracking results are consistently worse. However, for the highest observation probability the performance of both trackers is still good. As before, with lower observation probability the performance of uTrack drops faster than the performance of Biggles. In general Biggles has a better performance than uTrack.

**Fig 9:**
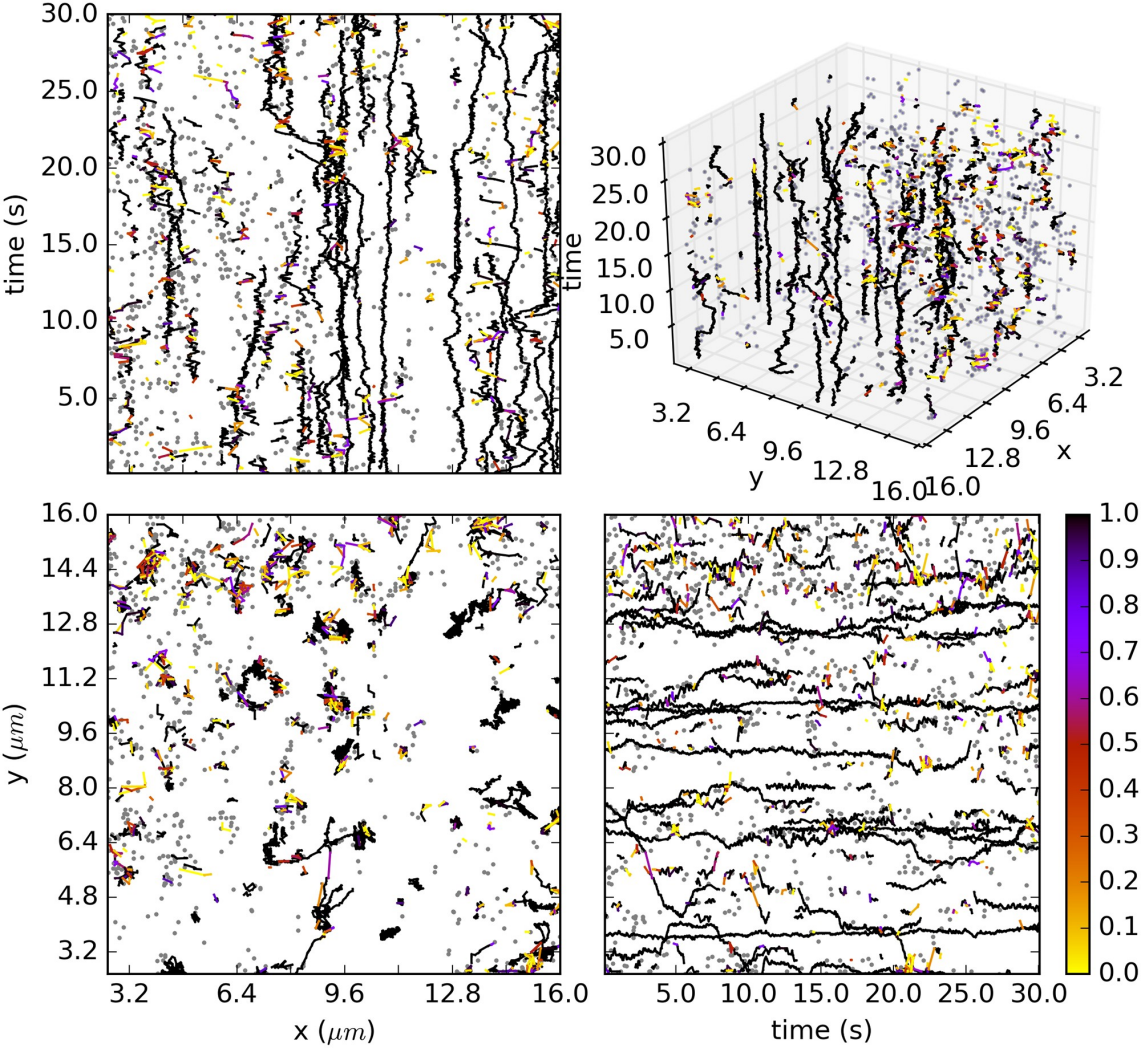
Two views on the tracking result for a SMM data set with 9761 observations. 4000 samples have been recorded. Links that appear in all samples are shown in blue. The colour of the other links indicates their frequency of occurrence as shown by the colour bar. Observations that are assigned to the clutter in all samples are shown as grey dots.

**Fig 10:**
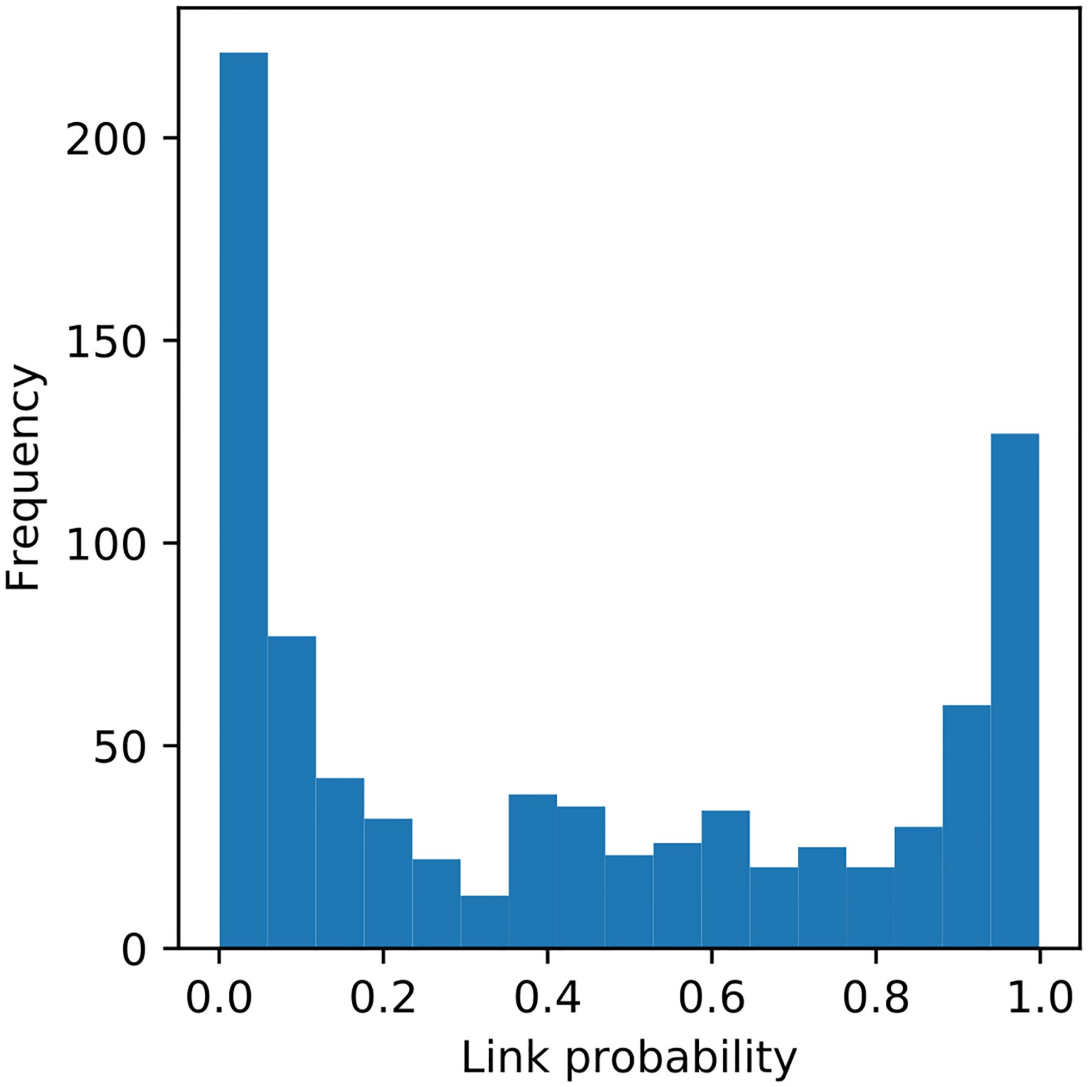
Histogram of the 850 links with estimated probability less than 1 for the data set in Fig 9. The number of links with $\hat{p}=1$ is 7923.

To illustrate Biggles we have chosen a typical SMM data set from our lab, which has been imaged for a co-localisation experiment. The data sets was acquired by total internal reflection fluorescence (TIRF) microscopy using organic dyes (enhanced green fluorescent protein) [33]. We imaged Epidermal Growth Factor (EGF) Dyomics 549-P1 on CHO cells stably expressing wild type EGF receptors at a level of about 50000 receptors per cell, transiently transfected with PLCd-PH-eGFP. The data set has a 16*μm*×16*μm* (100pixels×100pixels) field of view, in 30*s* 600 image frames have been acquired and 9761 observations have been detected using an in-house algorithm [34]. We recorded 4000 tracking samples. The result is shown in Fig 9. In total 8773 links occurred, of which 7923 appear in every sample and can be considered certain. The histogram of the 850 links with $\hat{p}<1$ is shown in Fig 10. The total number of samples drawn in both chains is 34 273 600.

The tracking result is plausible. All obvious tracks are found and there are no obviously wrong links with high probability. Biggles considers most of the tracks as uncontroversial. About 10% of the links do not appear in all samples. Most of those links have either a small probability or a high probability. We found 273 links (3.1%) with $0.2\leq \hat{p}\leq 0.8$, see Fig 10. Those links may indicate locally high crowding, track splitting or merging, large gaps between two track pieces and more. The final acceptance rate of the Metropolis-Hastings sampler is low, between 0.4% and 1.4% for the different move types (Table 1). Identity moves could be avoided for 5 of the move types. Relatively many identity moves occur for the birth type (5.3%) and the update-type (3.5%). However, for these moves we also observe below-average proposal rejections so that the acceptance rate is not affected.

**Table 1:** Statistics of the move types for one sampler chain. Data set SMM–9761.

move type	rejected	identity	accepted	total
Birth	94.3%	5.3%	0.4%	1 928 159
Death	99.6%	0.0%	0.4%	1 926 784
Extend	98.8%	0.0%	1.2%	1 927 725
Reduce	98.0%	0.8%	1.2%	1 924 570
Split	99.5%	0.0%	0.5%	1 926 823
Merge	99.1%	0.4%	0.5%	1 926 366
Update	95.1%	3.5%	1.4%	1 928 992
Transfer	98.8%	0.0%	1.2%	1 927 295
Cross-over	99.6%	0.0%	0.4%	1 926 086

## 4 Discussion

Biggles expands the concept of the single particle tracker by sampling the posterior distribution of possible tracking solutions and their governing parameters. Therefore, with Biggles we enable the calculation of errors and other descriptive statistics for tracking solutions. The set of all partitions does not come with an canonical distance measure, which is needed for some statistics. There are several possibilities to introduce a distance measure such as: treating the tracking solutions as graphs and employing the GED, or treating tracking solutions as vectors of a high dimensional space where each pair of linkable observations contributes one dimension. We used the GED as part of the convergence assessment.

The knowledge of the most likely solution remains of limited use as long as we do not know *how* certain the solution is and how likely alternative solutions are. With Biggles we have now the means to do what we would do in any other measurement process: evaluate the error on our solution.

Biggles does not need to input parameters that control the tracking process. On the contrary these parameters are a part of the solution. We show a example of parameter distributions in Fig 4. There are some slight biases in the recorded parameter samples. The design of the survival probability, *p*_*s*_, implies a exponential distribution for the track length. However, tracks of length 0 or 1 are not allowed and all tracks are limited to be no longer than the number of imaged frames. The samples stem in fact from a truncated exponential distribution. Since each observation is either explained as clutter or as observed track the biases in the clutter rate, λ_*c*_ and observation probability, *p*_*o*_, are opposing. However overall there is a very good agreement of the sampled parameters with those of the GTR, see Fig 4.

A direct validation of the Biggles samples is not easy since the ground truth *distribution* is not known. We treated the GTR links as if they would be certain under the given model, which is not true, since a particle can move in an unlikely manner. However, it should remain the exception that the ground truth is unlikely under the model, since the model shall explain the motion of the particles. In fact, we found very good agreement of the sampled links with the GTR. As expected in the case of higher track density we found more uncertainty in the links. This is not a shortcoming of the algorithm, but its point. In high crowding situations the track assignment is less clear and a higher temporal and spatial resolution would be required to achieve more confidence in a specific tracking solution. With Biggles we quantify our confidence in a specific solution and produce representative samples of alternatives. For the data sets in Fig 5 we found that for the vast majority of the cases links in the GTR and frequent links in recorded samples coincide. If we consider recall and precision as functions of the minimal estimated link probability, *Rec*(*p*_*min*_) and *Pre*(*p*_*min*_) respectively, we do expect to see *Rec*(*p*_*min*_) → 1 for *p*_*min*_ → 0 and *Pre*(*p*_*min*_) → 1 for *p*_*min*_ → 1. That means, we expect to find any real link in at least in one sample and we expect not to find the same false link in all samples. For the low density in Fig 5 this seems to be the case. For higher density data sets we still find *Rec*(0) > 0.99 and *Pre*(1) > 0.99.

**Fig 11:**
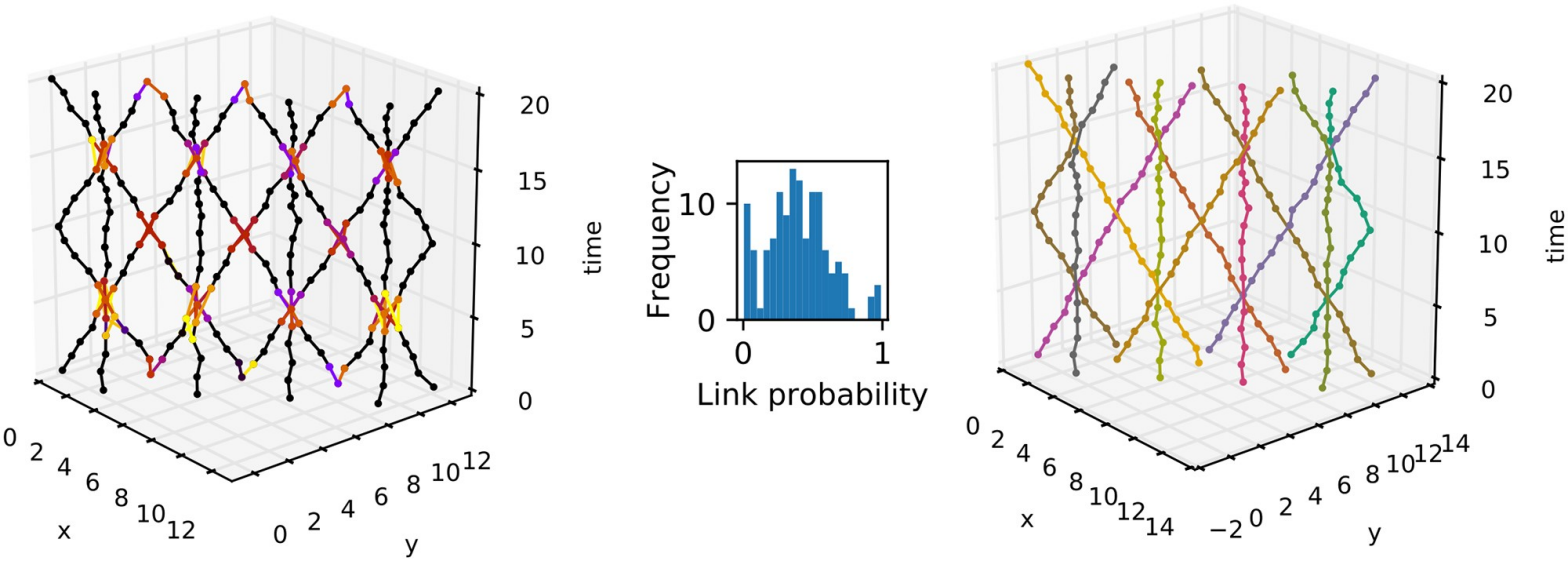
Tracking complicated data sets. The left panel shows the link probabilities. Certain links are in blue, highly probable links are in red, 50-50 links are coloured orange and unlikely links are in green. The middle panel shows the histogram of the links that are not certain, i.e. $\hat{p}\left(l\right)<1$. The right panel shows the maximum posterior solution.

Even though the definition of a track does not include merging or splitting of tracks, splits and merges are represented in Biggles sampling, see Fig 11 and S6 Fig. The data set we used to demonstrate this is composed of straight lines with some added white noise. At time points 5 and 15, there are four points where three lines intersect. At time point 10 there are three intersections of two lines and at time points 0 and 20 there are three points where two lines meet. Track splitting (or merging) events appear as regions with larger uncertainty in the links. In the course of the sampling, the observations at the intersection are assigned to different tracks. Fig 11 demonstrates also the tracking of directed tracks including change of direction. We made no special adjustments to track this data set.

Another approach to verify the results of Biggles uses derived quantities. We demonstrated this with the diffusion coefficient. Our results show that on average Biggles accurately reproduces the diffusion constant of the GTR. However for individual data sets the results may deviate from the GTR, significantly in terms of the sample standard deviation.

We have demonstrated in Fig 9 that Biggles can analyse real-world microscopy data sets. For large data sets the sampling process slows down from thousands of samples per second for small data sets to a few samples per second. The final sample rate for the SMM-9761 data set was about 500 samples per seconds using a fast commercial desktop computer. There are moderate improvements possible using faster computers, however software engineering of the code promises the most significant improvement. This concerns for example the handling of data of significant size, which effects the speed the algorithm and the number of samples that can be held in the chains. The sampling of the moves depends on internal book keeping to get a high efficiency in identifying viable proposals. Solutions for implementation problems are independent from the algorithm development and are not discussed here.

There is another possibility to improve performance for large data sets. Our SMM data sets can contain about 500 000 observations. We are developing a chunked version of the algorithm that divides the data into overlapping spatial chunks, tracks the observations in each chunk separately and reconciles the results. This approach will also allow the usage of computing clusters or cloud computing resources.

The sampling process still lacks efficiency. On one hand the track partition samples are correlated which reduces the effective sampling size. The correlation between the track samples lies in the nature of the Metropolis-Hastings proposals. The vast majority of links of the proposal will be identical with the links of the last sample. If *Q*_*m*_(*ω*, *ω*′) > 0, then the GED between *ω* and *ω*′ is at most 6 links, if *m* is any of the move types merge/split (1 link difference), transfer (6 links), cross-over (4 links), or update (max. 4 links). If the move type *m* is any of birth/death or extend/reduce then both partitions differ in one track only, with a GED less then *T* links. All moves at most modify 2 tracks at a time.

On the other hand, the acceptance rate of the sample recording of data set SMM–9761 is very low as we showed in Table 1. All moves are likely to propose changes to the partition which have a low target density. In many cases, we have *Q*(*ω*|*ω*′) ≫ 0 even if *P*(*ω*|*θ*, *Y*) ≪ *P*(*ω*′|*θ*, *Y*). If for example the update move is applied to a long track, then the update will be attempted at any time point with equal probability. However, often it is the case that there are only very few time points for which the move would produce an acceptable proposal, making the acceptance rate very low. In this regard the proposal mass function is wide in comparison with the target distribution. In the current implementation, Biggles therefore suffers from both a slow exploration speed and a low acceptance rate. Modifications to improve the proposal mass function both increase the complexity of the calculation of the proposal mass ratio and increase the complexity of the proposal creation itself due to the employment of smarter algorithms, extensive internal book keeping and so on.

The size of a single partition sample is linear in *N*. It is therefore not possible to keep a large number of samples in memory. This affects the number of chains that can be kept and their size, but also the number of samples that can be recorded. Especially for multimodal distributions it is important that the chains are long enough to cover the relevant part of Ω. If the chains are too short the assessment of convergence may go wrong. The assessment can go wrong in two ways; the chains have reached the stationary distribution, but are in different parts of it because they are too short to cover the whole support. It is also possible that the chains have not yet reached the stationary distribution, but accidentally it appears as such. The choice of the starting partition is therefore of some importance. Our approach runs two chains with the minimal partition *ω*^0^ and a maximal partition *ω*^*max*^ as starting points.

The huge size of Ω seems to imply than any practicable sample size is far too small to explore Ω. However, experience shows that the vast majority of links are uncontroversial with *p*(*l*) > 0.99. The interesting subset Ω* ⊂ Ω is much smaller, and often focuses on small spatio-temporal regions. If such regions are disconnected, they could be sampled separately, which further reduces the size of Ω*. Still, the size of Ω* can be substantial. It is very difficult to say how many samples are required to cover it and it depends on the data set.

## 5 Conclusion

We present in this paper a prototype of a novel approach to single particle tracking (SPT) that samples from the combined probability mass function/probability density function of the track partitions and its governing parameters. This enables not just the estimation of the most likely tracking solution but also provides us with a measure of uncertainty of this solution and likely alternative solutions. Thus Biggles normalises SPT with standard measurements that provide measured value and error estimate. Our approach also has the potential be used to estimate the error on derived quantities as we demonstrated on the diffusion rate. The algorithm can handle different condition without special adjustment, such as random walk, directed motion, change of direction and track branching. We demonstrated that Biggles can analyse data sets with about 10 000 observations. The implementation of Biggles is complex. Smarter algorithms, optimised convergence control and sample recording and professional software engineering will improve the performance of the algorithm. We also indicated other potential improvements. Biggles opens a new direction in SPT.

## Supporting information

S1 Fig Recovering of the simulation parameters. The Q-Q plots of the parameter samples for the GTR and the parameter samples created by Biggles. Shown are a series of ten data sets with low track density(top) and a series of data sets withhigh track density (bottom).
(PNG)

S2 Fig Biggles tracking example. A mixture between directed motion and random walks. Tracks have a chance to change the mode of motion. The grey dots mark the clutter observations.
(PNG)

S3 Fig Biggles tracking example. A mixture between directed motion and random walks. Tracks have a chance to change the mode of motion. The grey dots mark the clutter observations.
(PNG)

S4 Fig Biggles tracking example. A 50-50 mixture between random walks with two different diffusion coefficients, *d*_1_ = 0.45*pix*/*fr* and *d*_2_ = 0.9*pix*/*fr*. Each track has one mode of mode of motion.
(PNG)

S5 Fig Biggles tracking example. Random walks with regions of different densities. Each track has one mode of mode of motion.
(PNG)

S6 Fig Alternative views of the complicated data set. Shown are the link probabilities.
(PNG)

S7 Fig Example of observation and estimated states of a track. (PNG)

S8 Fig Dependency on the process noise. A step length of 1*pixel*/*frame* is equivalent to *D* ≈ 0.26*μm*^2^/*s* at a pixel size of 160*nm* and a frame rate of 20*Hz*.
(PNG)

S1 Appendix Algorithm notes. Detailed information about the algorithm, discussions of properties of the space of valid track partitions Ω and proof that the partition sampler is ergodic.
(PDF)
