# Supplementary figures and images for: A global sampler of single particle tracking solutions for single molecule microscopy
Source: PLoS One. 2019 Oct 28;14(10):e0221865. doi: 10.1371/journal.pone.0221865 (PMC6816549; doi:10.1371/journal.pone.0221865)

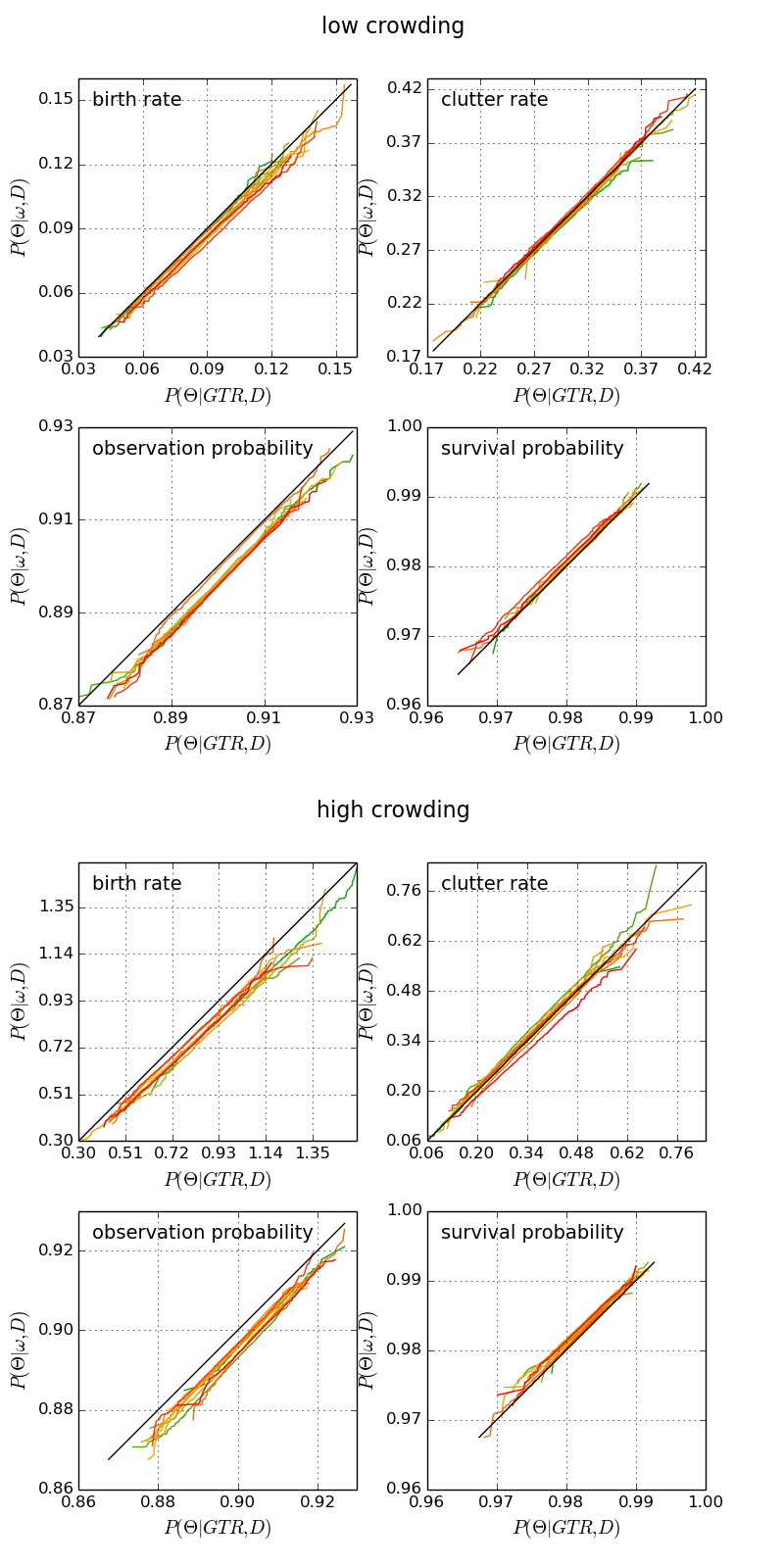

Supplement: S1 Fig — The Q-Q plots of the parameter samples for the GTR and the parameter samples created by Biggles. Shown are a series of ten data sets with low track density(top) and a series of data sets withhigh track density (bottom). (PNG) [file pone.0221865.s001.png]

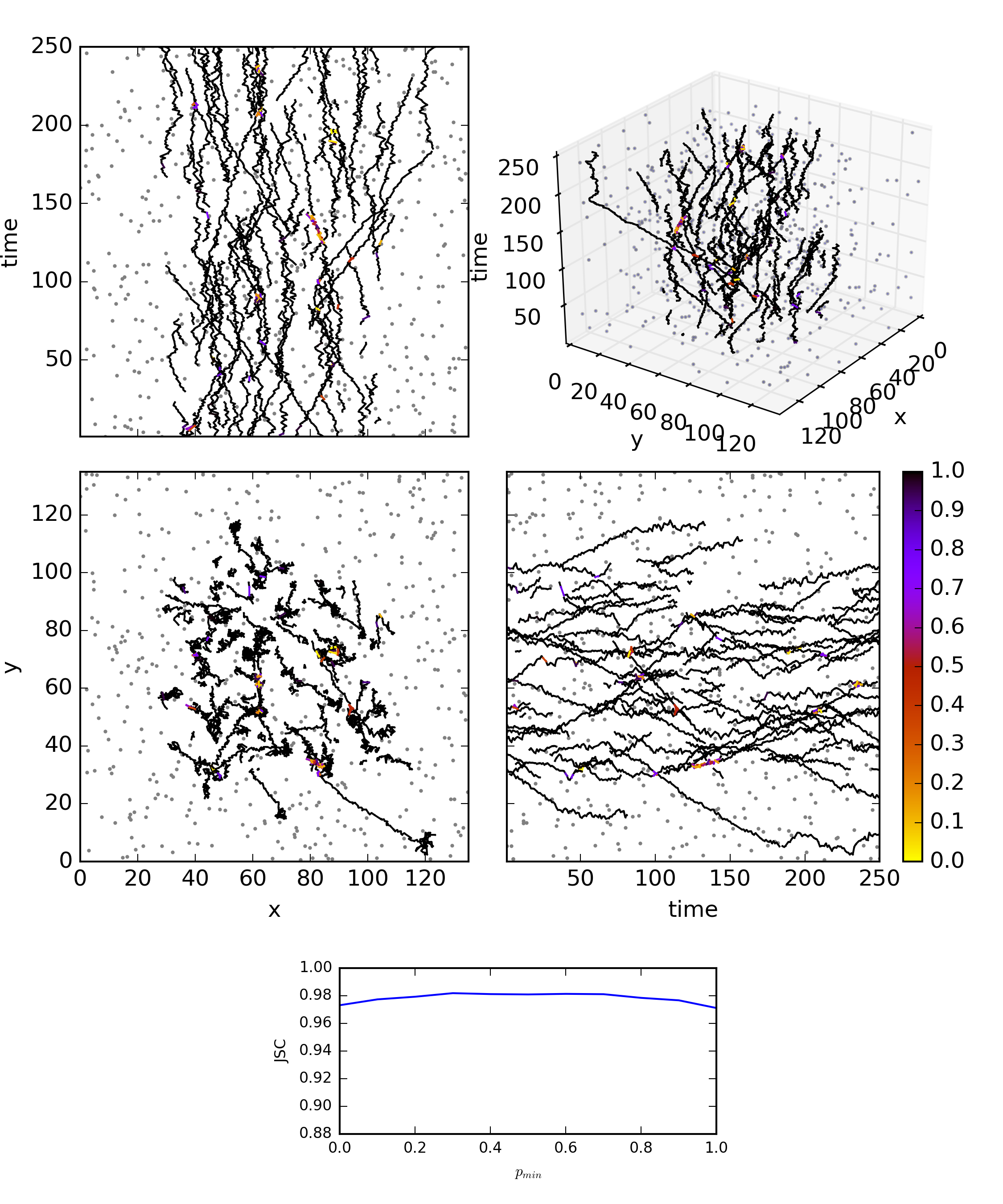

Supplement: S2 Fig — A mixture between directed motion and random walks. Tracks have a chance to change the mode of motion. The grey dots mark the clutter observations. (PNG) [file pone.0221865.s002.png]

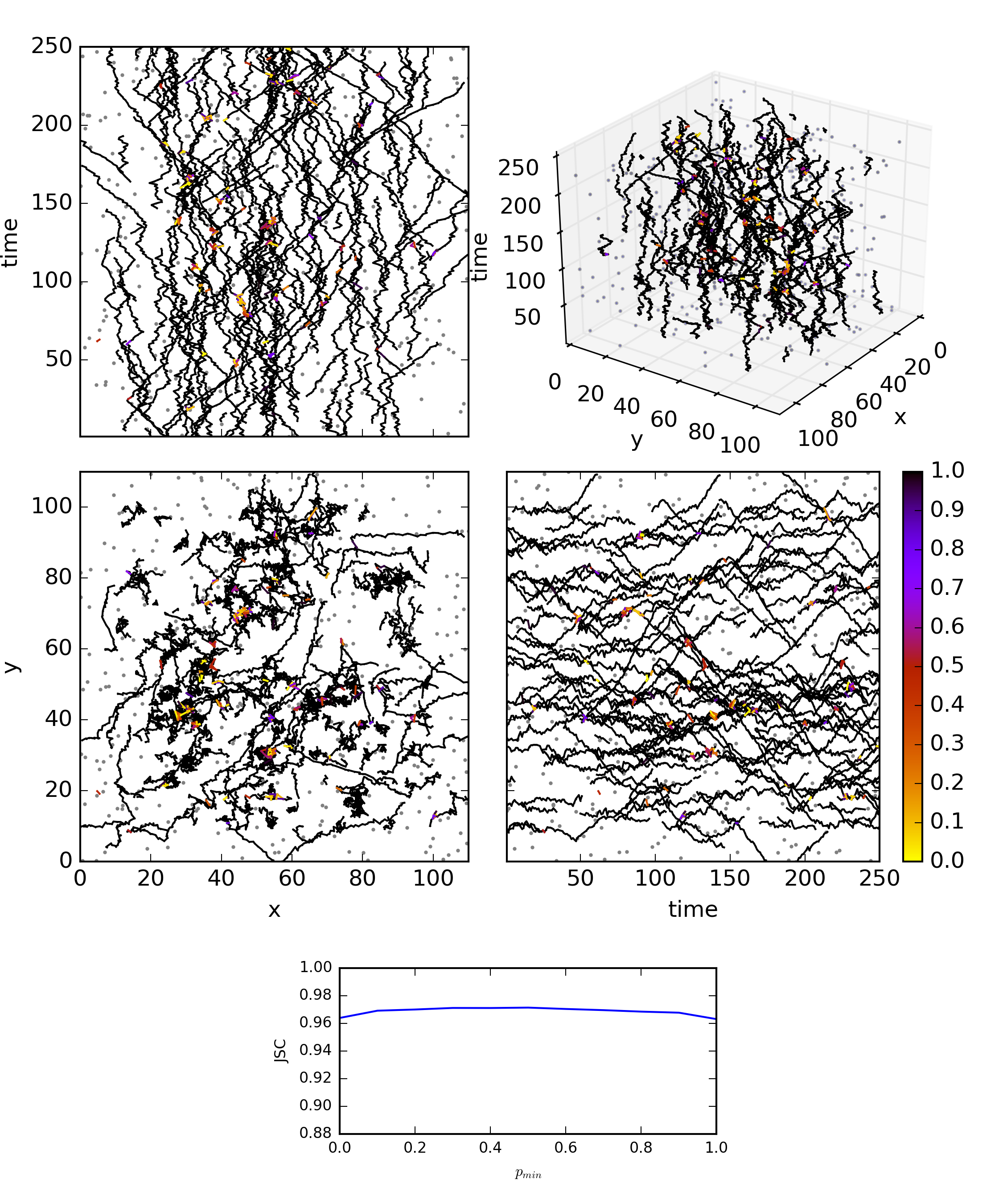

Supplement: S3 Fig — A mixture between directed motion and random walks. Tracks have a chance to change the mode of motion. The grey dots mark the clutter observations. (PNG) [file pone.0221865.s003.png]

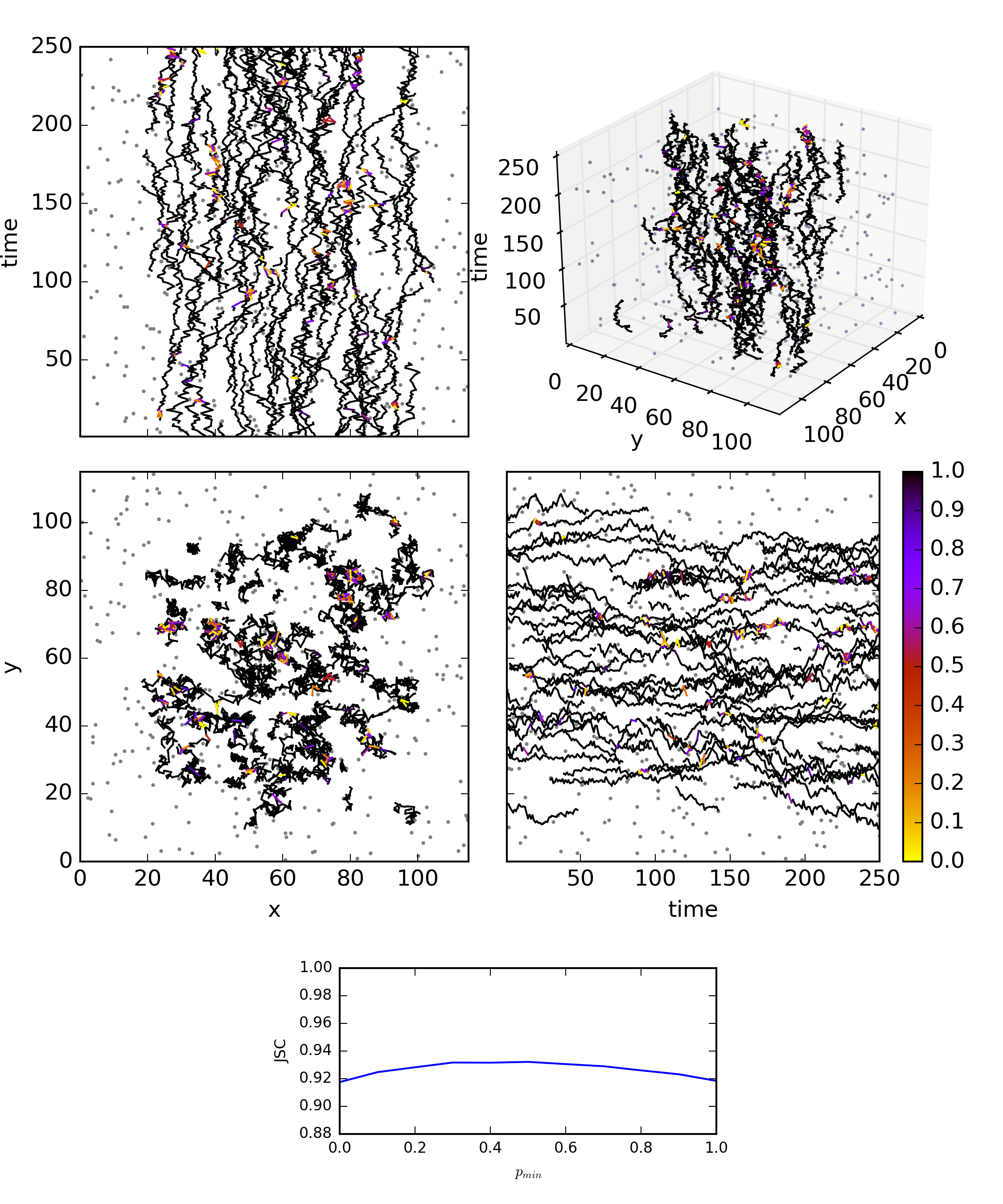

Supplement: S4 Fig — A 50-50 mixture between random walks with two different diffusion coefficients, d1 = 0.45pix/fr and d2 = 0.9pix/fr. Each track has one mode of mode of motion. (PNG) [file pone.0221865.s004.png]

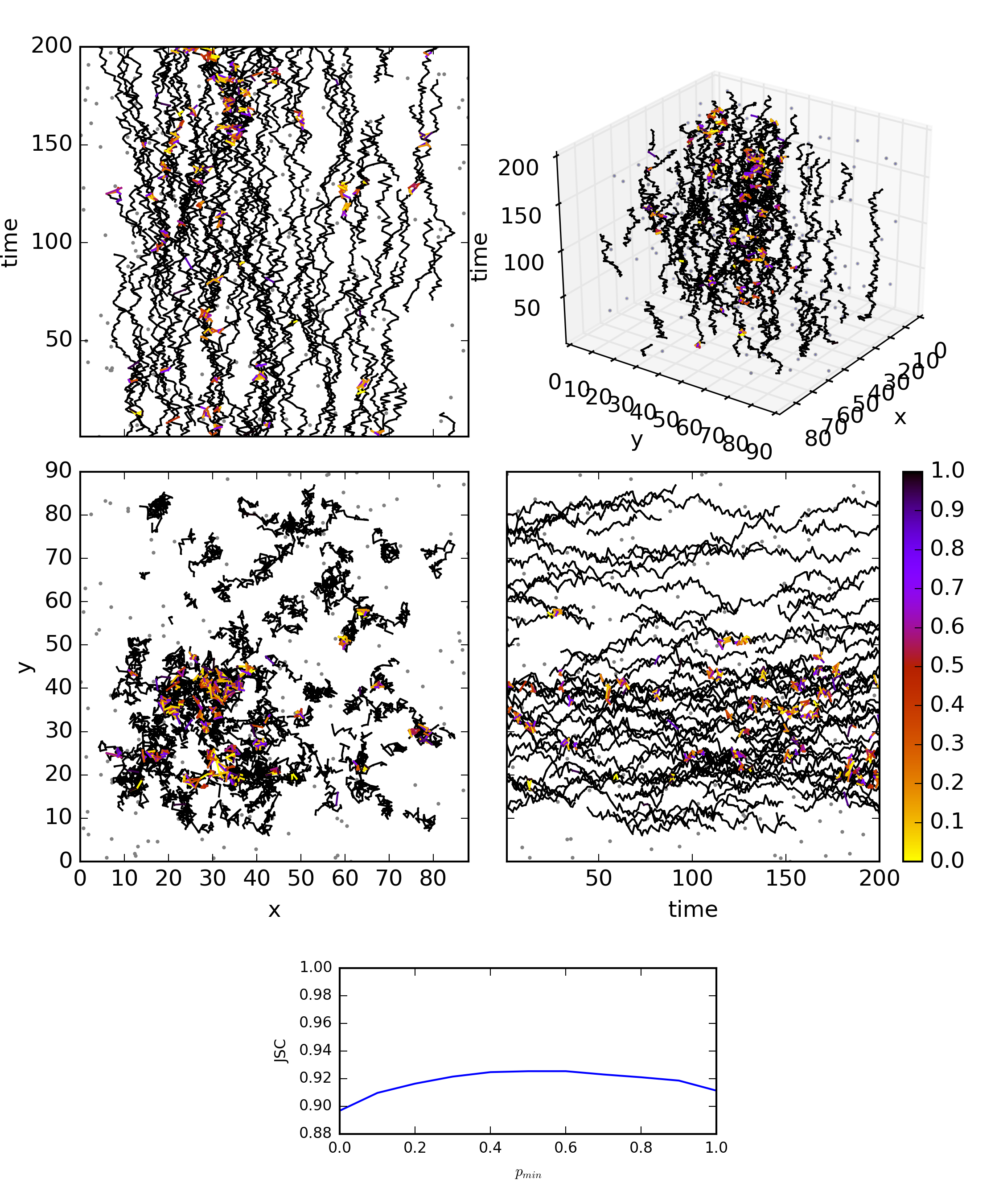

Supplement: S5 Fig — Random walks with regions of different densities. Each track has one mode of mode of motion. (PNG) [file pone.0221865.s005.png]

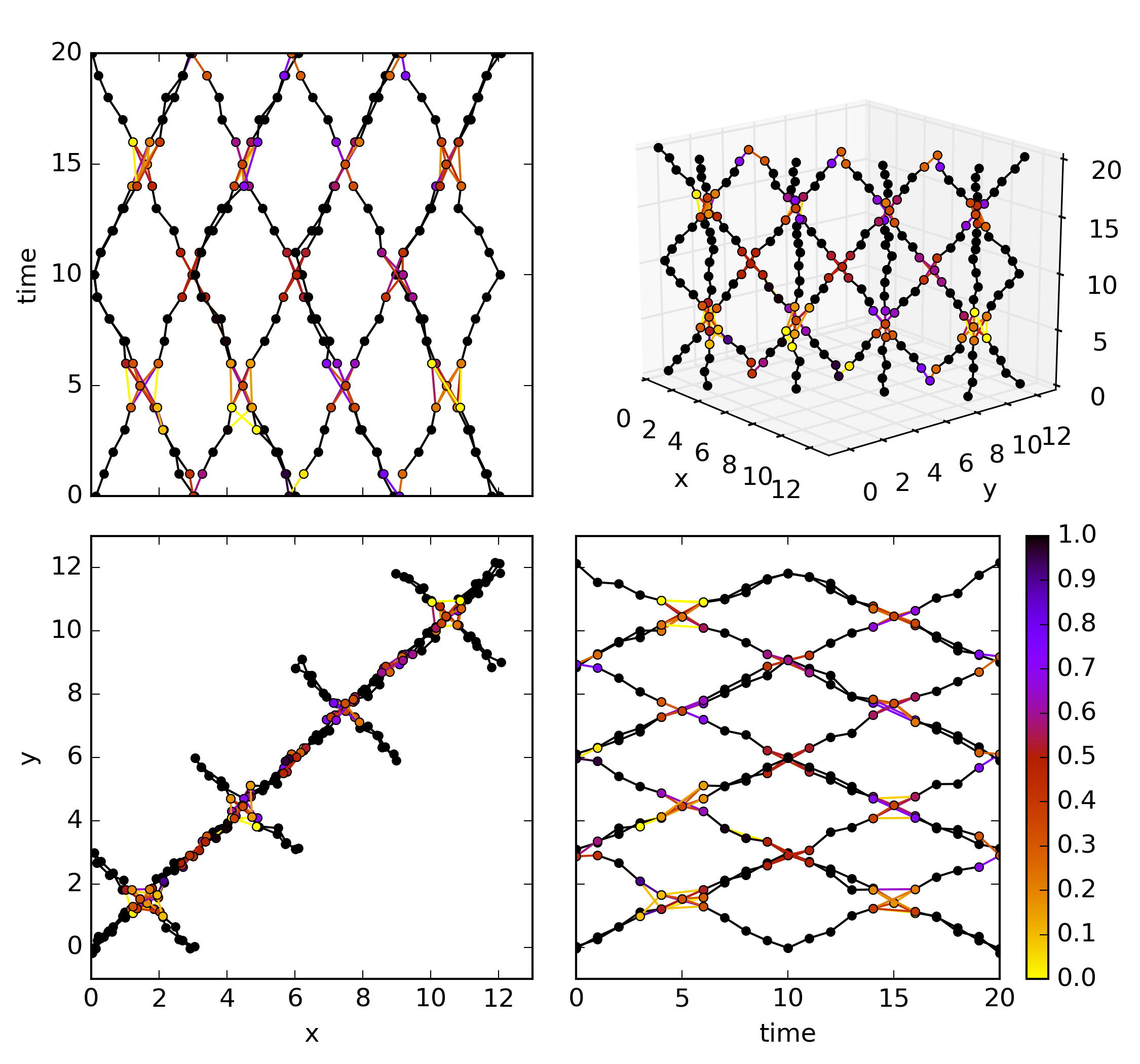

Supplement: S6 Fig — Shown are the link probabilities. (PNG) [file pone.0221865.s006.png]

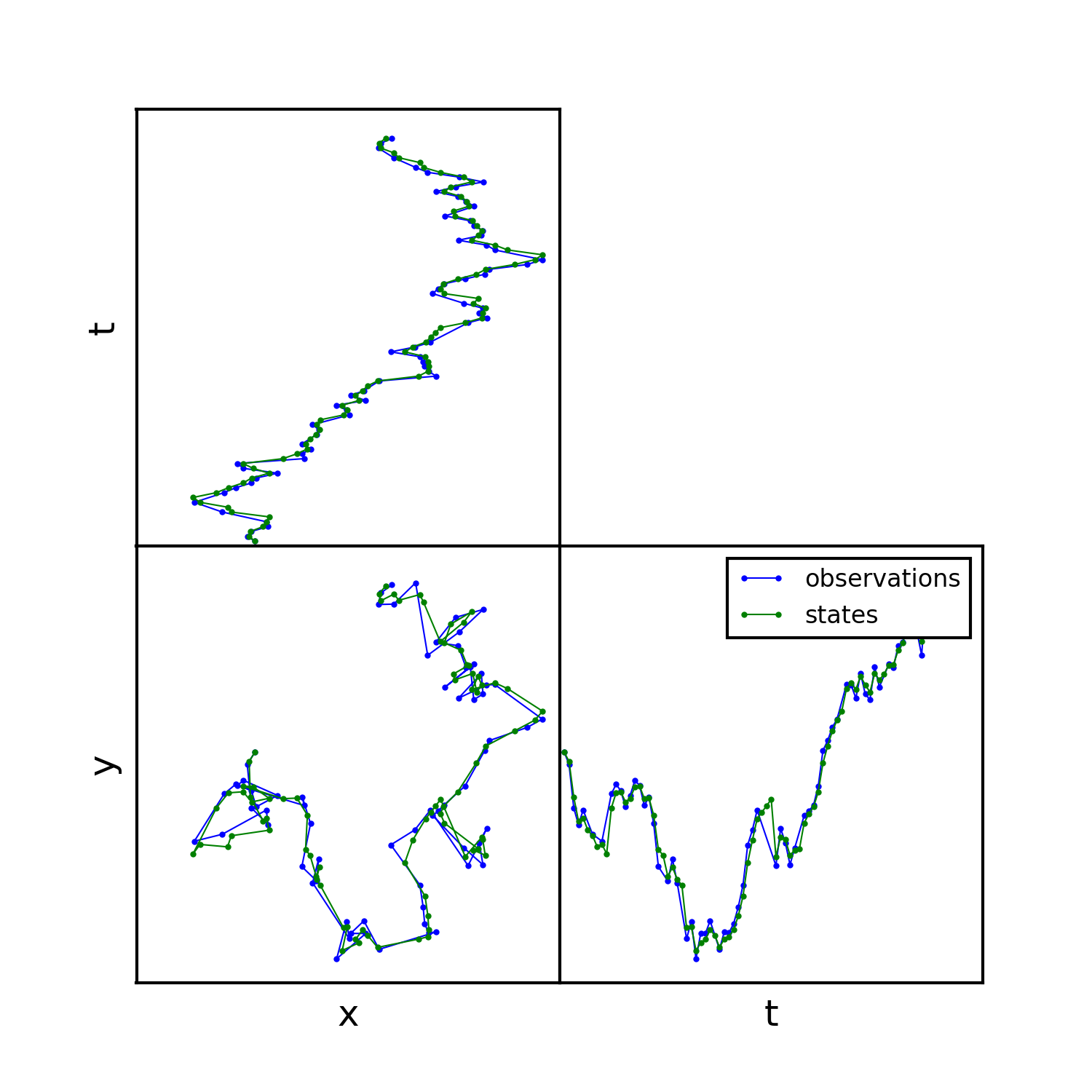

Supplement: S7 Fig — (PNG) [file pone.0221865.s007.png]

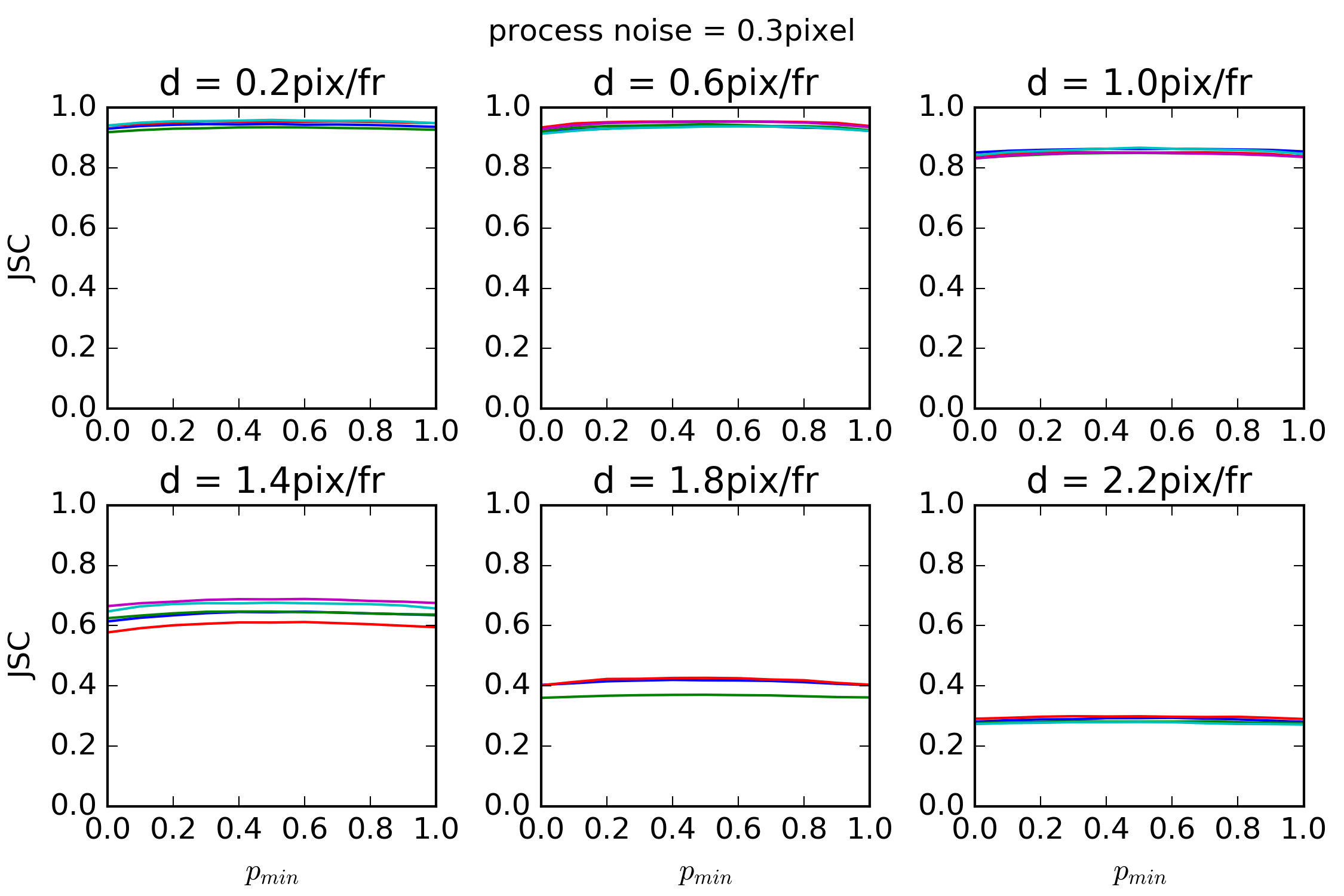

Supplement: S8 Fig — A step length of 1pixel/frame is equivalent to D ≈ 0.26μm2/s at a pixel size of 160nm and a frame rate of 20Hz. (PNG) [file pone.0221865.s008.png]
