# Supplementary material for: A global sampler of single particle tracking solutions for single molecule microscopy
Source: PLoS One. 2019 Oct 28;14(10):e0221865. doi: 10.1371/journal.pone.0221865 (PMC6816549; doi:10.1371/journal.pone.0221865)
Supplement: S1 Appendix — Detailed information about the algorithm, discussions of properties of the space of valid track partitions Ω and proof that the partition sampler is ergodic. (PDF) [file pone.0221865.s009.pdf]

A global sampler of single particle tracking solutions for single molecule microscopy

S1 Appendix

Contents

1 Observations, tracks and clutter **2**

2 Gibbs sampler **2**

3 Metropolis-Hastings Sampling from the track partition **3**
- 3.1 Partition conditional probability 6
- 3.2 Kalman filter as time evolution model 7
- 3.3 Track observations likelihood 9
- 3.4 Clutter observations likelihood 9
- 3.5 Parameter prior 10

4 Sampling the parameters **10**

5 Notes on partition distances, ergodicity and move design **10**
- 5.1 Distances between partitions 11
- 5.2 Distance based on Biggles moves 11
- 5.3 Ergodicity 13
- 5.4 Number of modes of the target distribution and move design 13

6 Technical Notes **14**
- 6.1 Diffusion coefficient 14

1 Observations, tracks and clutter

Single particle tracker track *particles* such as molecules. The appearance of a particle in an image (or other measurement) is a feature of that image. Biggles takes as input a set of features specified as spatial x, y and temporal t coordinates. These correspond to the output of a feature detector and are collectively called *observations*, $Y = \{Y_t = [x, y]^T\}$, of a set of particles. We assume that the time lag between images is constant, so that $t \in \{1, \ldots, T\}$, where T is the number of image frames taken. This is not a limitation of the algorithm, but it makes implementation easier. Missing frames mean that there are no observations at the time points in question. The set of observations that have all been generated by the same particle are called a *track*. Observations may be deemed to have arisen spuriously from the feature detector and not from a particle of interest. Spurious observations are collectively termed the *clutter*, $k_0 \equiv Y^0$. The subset of observations assigned to track k_i, $i = 1 \ldots K$, is Y^i, We have $Y^i \cap Y^j = \emptyset$ if $i \neq j$ and $\cup_{i=0}^{K} Y^i = Y$. Because of these properties, the set of all tracks and the clutter is termed a (track) *partition*, $\omega = \{k_0, \ldots, k_K\}$, of the input, where ω contains K tracks and the clutter k_0.

Condition 1 *A track fulfils the following conditions:*

1. *a track has at most one observation at each time point,*
2. *the number of observations of the track is at least 2,*
3. *the spatial distance of any two observations in a track has a limit that linearly depends on the time lag between the observations.*

A track can be written as

$$k_i = (t_0^i, t_1^i, Y^i),$$

where t_0^i and t_1^i are the first and the last time point of the track respective. This also is the computational representation of a track. It is allowed that first and last time points do not have observations. The length of the track is the number of its time points, $t_1^i - t_0^i + 1$. With condition 1.2 follows that the length of any track is in $[2, T]$. The third condition means that a maximum velocity of the particle is assumed.

2 Gibbs sampler

Our aim is to explore the distribution $P(\omega, \theta|Y)$, where ω is a partition, θ is a set of model parameters and Y are the observations. Internally, the parameters θ are represented by a tuple containing floating point values and one matrix: $(\lambda_b, \lambda_c, p_s, p_o, R)$ where

- λ_b: the mean number of new tracks appearing per frame and area.
- λ_c: the mean number of clutter observations per frame and area.
- p_s: the probability that a track will survive from frame t to $t + 1$, i.e. p(“track present at $t + 1$” | “track present at t”).
- p_o: the probability that a track will generate an observation at a time point.
- R: a 2×2 matrix giving the assumed covariance of observation error of the Kalman filter (section 3.2).

We use a Gibbs sampler to avoid having to sample from $P(\omega, \theta|Y)$ directly. We draw a train of samples ω_i, θ_i by alternately drawing them from the two conditional distributions:

$$\omega_i \sim P(\omega|\theta_{i-1}, Y) \quad \text{and then} \quad \theta_i \sim P(\theta|\omega_i, Y),$$

where ω_0 and θ_0 are initialised to some values. In principle we are free to choose ω_0 and θ_0. Practically we avoid extreme values for θ_0 and fix it to uncontroversial moderate values. We are running two chains and initialises ω_0 in the first chain to a state where all features are assumed to be spurious detections. The second chain initialises ω_0 to a maximum partition, which is created by a greedy algorithm. The method how the maximum partition is created is not critical. In principal it works as follows:

1. Group and sort the available observations by time and start with the earliest group,
2. pick a random observation from the current time group and use it as first observation of a new track,
3. try to randomly add a new observation to the track from the time group just after the current last observation of the track, respecting condition 1.2 and allowing a gap of up to 5 time points,
4. repeat step 3 until its options are exhausted,
5. if the resulting track has more than one observations, save it and remove its observations from their time groups,
6. go to step 2, if the current time group is empty, pick the next time group, if all time groups are used, finish,
7. assign all remaining observations to the clutter.

Thus the Gibbs sampler alternates between two steps:

1. Sampling from the track partition using the Metropolis-Hastings algorithm (section 3)
2. Sampling the parameters (section 4)

3 Metropolis-Hastings Sampling from the track partition

The heart of the current implementation is a Metropolis-Hastings sampler. The target partition of the sampler is given by $P(\omega|\theta, Y)$ and a new partition, ω^*, is sampled from the proposal partition $Q(\omega^*|\omega)$. The proposal density Q is given by a set of moves that transform ω to ω^* and their probability to be executed.

When proposing ω^* from ω we choose uniformly from one of seven different types of move. In the following description, a “no observation” is an item that represents a time point of a track without observation. All moves enforce the formal constraints for tracks given in definition 1.

- Birth: Two time points are randomly chosen as first and last time point. Beginning with the first, for each time point a list of all candidate observations is assembled each with a weight of 1. A “no observation” is added to the list with the weight of 1/10. An element of the list is randomly sampled and added to the track. This moves increases the number of tracks by 1 and reduces the number of clutter observations by at least 2.

- Death: a track is chosen uniformly and at random from the partition and is demoted to the clutter. This move reduces the number of tracks by 1 and increases the number of clutter observations by at least 2.
- Extend: a track is chosen uniformly and at random from the partition. It is chosen uniformly and at random in which temporal direction the track is to be extended and the track is extended as in the birth move. This move increases the length of the track by at least 1, it may or may not add observations to the track and hence may or may not reduce the number of clutter observations.
- Reduce: a track is chosen uniformly and at random from the partition. It is chosen uniformly and at random in which temporal direction the track is to be truncated and a time point within it is sampled uniformly and at random. The portion of the track extending from the sampled time point in the sampled direction is demoted to the clutter. This move will reduce the length of the track by at least 1, it may or may not reduce the number of observations of the track and add those observations to the clutter.
- Merge: a pair of nearby but non-temporally-overlapping tracks are sampled uniformly and at random from all such pairs and are merged into one by simple concatenation. This move reduces the number of tracks by 1, the clutter remains unaffected. The length of the new track is larger than or equal to the sum of the lengths of the tracks merged. The number of observations of the new track is equal to the sum of the number of observations of the merged tracks.
- Split: a track is chosen uniformly and at random from the partition. An time point is sampled uniformly and at random from within the track and the track is split at this point into two new tracks. The earlier track is randomly shortened by 0 or more final time points without observations. This moves increases the number of tracks by 1, the clutter remains unaffected. The sum of the lengths of the new tracks is less than or equal to the length of the split track. The sum of the observations of the new tracks is equal to the number of observations of the split track.
- Update: a track is chosen uniformly and at random from the partition. A time point is sampled uniformly and at random from the time span of the track. The track may or may not have an observation at the sampled time point. A new observation is sampled uniformly and at random from the set of all spatially nearby observations plus a "no observation". The original observation is demoted to clutter and the sampled observation (if any) is included into the track. This moves leaves the number of tracks and the length of the updated track unaffected. The number of observations of the updated track changes by -1 ("drop"), 0 ("swap") or 1 ("pick-up"), the size of the clutter changes by 1 (drop), 0 (swap), -1 (pick-up). Even in the swap case, the observations of the updated track and the clutter are changed. Any empty swap is prevented.
- Transfer: a pair of tracks is chosen from the set tracks that permit that move at one or more time points. One track is the donor the other track is the acceptor. A time point is uniformly sampled from the set of time points at which a valid transfer is possible. The observation of the donor at this time point is removed and added to the acceptor. This move leaves clutter, number of tracks and track lengths unaffected. The number of observations of the donor is reduced by 1 and the number of observations of the acceptor is increased by 1.

- Cross-over: a pair of tracks is chosen from the set tracks that permit that move at one or more time points. A time point is uniformly sampled from the set of time points at which a valid cross-over is possible. Both tracks are split into two branches at the time point so that each branch has at least one observation. Two new tracks are formed each using one branch of each old track. If the old tracks are denoted by (a_1, a_2) and (b_1, b_2) than the new tracks are denoted by (a_1, b_2) and (b_1, a_2). This move leaves the number of tracks and the clutter unaffected. The sum of the length of the new tracks is equal to the sum of the lengths of the old tracks.

There are 6 pairs of moves that can undo each other: birth – death, extend – reduce, merge – split, update – update, transfer – transfer and cross-over – cross-over. From a given partition ω any new proposal, ω^*, can be achieved by one move type only, e.g. a merge move can never have the same result as a extend move or birth move. The moves were so chosen such that the appropriate proposal density, $Q(\omega^*|\omega)$, is straightforward to compute. For example, given a birth move, $Q(\omega^*|\omega)$ is the likelihood of the birth that leads from ω to ω^*, while $Q(\omega|\omega^*)$ is the probability of the death that leads from ω^* to ω. In some cases a move does not create a valid partition. Example 1: A birth move has proposed a track that only contains 1 observation. Example 2: A reduce move is attempted but all tracks have length 2. Such cases are treated as $\omega^* = \omega$. Hence $Q(\omega|\omega) > 0$. However, efforts have been made to keep $Q(\omega|\omega)$ small.

We wish to find the mode of the posterior distribution $P(\omega|\theta, Y)$. We can re-arrange this posterior using the law of conditional probability which states $P(\omega|\theta, Y) = P(\omega, \theta|Y)/P(\theta|Y)$ combined with Bayes' theorem:

$$P(\omega, \theta|Y) = \frac{P(Y|\omega, \theta)P(\omega, \theta)}{P(Y)} = \frac{P(Y|\omega, \theta)P(\omega|\theta)P(\theta)}{P(Y)}$$

and hence

$$P(\omega|\theta, Y) = \frac{P(Y|\omega, \theta)P(\omega|\theta)P(\theta)}{P(\theta|Y)P(Y)} = \frac{P(Y|\omega, \theta)P(\omega|\theta)P(\theta)}{P(\theta, Y)}.$$

Since we use the Metropolis-Hastings algorithm which requires only a value proportional to this density. Removing terms independent of ω we obtain:

$$P(\omega|\theta, Y) \propto P(Y|\omega, \theta)P(\omega|\theta)P(\theta).$$

The $P(\theta)$ term is simply the prior on the model parameters. The $P(\omega|\theta)$ term is the likelihood of the tracks (the grouping of observations), independent of the physical properties (position, velocity and their errors) of the observations within them. This is a function of the model parameters and birth and death times for the tracks only. Since the data is partitioned between tracks and the clutter with no overlap, we may factorise the data likelihood term as follows:

$$P(Y|\omega, \theta) = P(Y^0|k_0, \theta) \prod_{i=1}^{K} P(Y^i|k_i, \theta)$$

where Y^0 is used to represent the clutter observations and $Y^1, ..., Y^K$ are the K sets of observations corresponding to the K tracks in the partition. $P(Y^0|\theta)$ is the likelihood of having seen the clutter observations, if we have given the parameters. $P(Y^i|k_i, \theta)$ is the likelihood of track k_i having generated the data we saw. Together we have

$$P(\omega|\theta, Y) \propto P(Y^0|k_0, \theta) \prod_{i=1}^{K} P(Y^i|k_i, \theta)P(\omega|\theta)P(\theta). \tag{1}$$

Taking logarithms, we obtain the final log density function used within the Metropolis-Hastings sampler:

$$\ell(\omega|\theta, Y) = \kappa + \ell(Y^0|k_0, \theta) + \sum_{i=1}^{K} \ell(Y^i|k_i, \theta) + \ell(\omega|\theta) + \ell(\theta) \tag{2}$$

where $\ell(\cdot)$ is used to denote the logarithm of probability density function (PDF) and κ is some arbitrary normalising offset. Thus the PDF has the following components:

- the partition conditional probability $P(\omega|\theta)$, section 3.1
- the track observations likelihood $P(Y^i|k_i, \theta)$, section 3.3
- the clutter observations likelihood $P(Y^0|k_0, \theta)$, section 3.4
- the parameter priors $P(\theta)$, section 3.5

The calculation of the priors $P(\theta)$ is not necessary for the acceptance probability since they does not depend on the partition and the two occurrences cancel each other.

3.1 Partition conditional probability

In this section we discuss evaluation of $P(\omega|\theta)$, that is the probability of the track partition given the model parameters. The physical properties of the observations, notably their position is not taken into account, only their grouping into tracks and clutter. We have no reason to assume a specific mutual dependency of the model parameters, we therefore may factorise the PMF as follows:

$$P(\omega|\theta) \propto P(\omega|p_s)P(\omega|p_o)P(\omega|\lambda_b)P(\omega|\lambda_c).$$

Note that $P(\omega)$ does not depend on R, since R has no bearing on the observation *assignment*.

We recall that Ω is the set of all possible partitions. Partitions only differ with each other in the assignment of observations to tracks and clutter. Each observation has a label, exchanging observations between tracks will result in a different partition. There is only one way (if any) to combine a given number of observations to a track. Reordering the observations in the clutter does not change the partition. Reordering of tracks does not change the partition. Each track is uniquely identified by the observations it contains and the first and last time point. The partition probability given the survival probability is the product of the probability of each survival and death. It has the general form of a binomial distribution, but does not feature the binomial coefficient. For a binomial distribution the sequences $(0, 1, 1)$ and $(1, 0, 1)$ mean both “2 out of 3”, while for a partition it matters which track survived. Similarly, the partition probability given the observation probability does care about which observations have been made. It has therefore the same form. The birth rate and clutter rate dependencies are modelled by Poisson distributions.

Each of these terms may defined in terms of the following values:

- N_t^t : the number of tracks present at t;
- N_t^s : the number of tracks that survive from $t-1$ to t ;
- N_t^b : the number of tracks that newly appeared at t;
- N_t^o : the number of observations assigned to a track at t;
- N_t^c : the number of observations deemed spurious at t.

The numbers are counted from ω. The number of track that survive at t is given by: $N_t^s = N_t^t - N_t^b$. Each of the individual parameter PDF terms can be written based on the definition of the corresponding parameters:

- $P(\omega|p_s) = \prod_{t=2}^{T} p_s^{N_t^s}(1-p_s)^{N_{t-1}^t - N_t^s}$;
- $P(\omega|p_o) = \prod_{t=1}^{T} p_o^{N_t^o}(1-p_o)^{N_t^t - N_t^o}$;
- $P(\omega|\lambda_b) = \prod_{t=1}^{T} \mathcal{P}(N_t^b; \lambda_b)$;
- $P(\omega|\lambda_c) = \prod_{t=1}^{T} \mathcal{P}(N_t^c; \lambda_c)$,

where $\mathcal{P}(x; \lambda)$ is the probability mass function of the Poisson distribution with mean λ evaluated at x and $N_t^y = N_t^o + N_t^c$. The corresponding log terms become:

$$\ell(\omega|p_s) = \sum_{t=2}^{T} N_t^s \log(p_s) + (N_{t-1}^t - N_t^s)\log(1-p_s) \tag{3}$$

$$\ell(\omega|p_o) = \sum_{t=1}^{T} N_t^o \log(p_o) + (N_t^t - N_t^o)\log(1-p_o) \tag{4}$$

$$\ell(\omega|\lambda_b) = \sum_{t=1}^{T} N_t^b \log(\lambda_b) - \lambda_b - \log(\Gamma(1+N_t^b)) \tag{5}$$

$$\ell(\omega|\lambda_c) = \sum_{t=1}^{T} N_t^c \log(\lambda_c) - \lambda_c - \log(\Gamma(1+N_t^c)) \tag{6}$$

3.2 Kalman filter as time evolution model

We use a Kalman filter with a Rauch-Tung-Striebel backwards smoothing filter to estimate the underlying states of the particles which generated the observed features so that the track posterior may be evaluated.

The state estimation consists of a prediction step that is based on the physical model followed by an update step that compares the prediction to the observation. In our system, the state is the position and the instantaneous velocity of a particle:

$$X = [x, \dot{x}, y, \dot{y}]^T,$$

where

$$\dot{x} = \frac{dx}{dt}, \quad \dot{y} = \frac{dy}{dt}$$

States evolve using first order dynamics:

$$X_t = AX_{t-1} + \boldsymbol{w}_t, \quad A = \begin{bmatrix} 1 & 1 & 0 & 0 \\ 0 & 1 & 0 & 0 \\ 0 & 0 & 1 & 1 \\ 0 & 0 & 0 & 1 \end{bmatrix},$$

where $\boldsymbol{w}_t \sim \mathcal{N}(0, Q)$. The observation of the particle is modelled by

$$Y_t = BX_t + \boldsymbol{v}_t, \quad B = \begin{bmatrix} 1 & 0 & 0 & 0 \\ 0 & 0 & 1 & 0 \end{bmatrix},$$

where $Y \in \mathbb{R}^2$ is the vector of the observed position, B is the observation model and $\boldsymbol{v}_t \sim \mathcal{N}(0, R)$ is the observation noise. The covariance matrix R is internally treated as a part of of the model parameters that are sampled by the Gibbs sampler (see section 4).

In the prediction step, the current estimate of the hidden state at time index t, $\hat{X}_t$, is evolved via the state evolution matrix A. In the update step, this estimate is refined by using any observations made. In this implementation the lack of an observation causes this update step to be skipped and the refined estimate is assumed to be equal to the prediction.

The prediction step is represented by the following recurrence relations:

$$\hat{X}_{t|t-1} = A\hat{X}_{t-1|t-1}, \quad P_{t|t-1} = AP_{t-1|t-1}A^T + Q$$

where $\hat{X}_{t-1|t-1}$ is the state estimate after the update step at time point $t-1$ and $\hat{X}_{t|t-1}$ is the state estimate at time t after the prediction step, but before the update step. $P_{t|t-1}$ and $P_{t|t}$ are, respectively, our prediction of the state estimation error and our refined prediction of the state estimation error.

The updated state at time t, $\hat{X}_{t|t}$, is represented by the following recurrence relations:

$$\hat{X}_{t|t} = \hat{X}_{t|t-1} + K_t\tilde{Z}_t, \quad P_{t|t} = (I - K_tB)P_{t|t-1}$$

where

$$\begin{aligned}
\tilde{Z}_t &= Y_t - B\hat{X}_{t|t-1}, \quad \text{(Innovation)} \\
K_t &= P_{t|t-1}B^TS_t^{-1}, \quad \text{(Kalman gain)} \\
S_t &= BP_{t|t-1}B^T + R. \quad \text{(Innovation covariance)}
\end{aligned}$$

Here Y_t is the observed position of feature at time t and $\tilde{Z}_t$ is the residual with respect to the predicted feature position (innovation). We initialise the filter by choosing some arbitrary initial state estimate, $\hat{X}_{0|0}$, and setting the initial state covariance matrix, $P_{0|0}$, to some sufficiently large multiple of I so as to specify almost no certainty on the initial estimate. We use the recurrence relations to compute estimates of states and estimation error covariances up until the last time index for the track.

The covariance matrix of the process noise Q is set to the following by default:

$$Q = \begin{bmatrix} 0.3^2 & 0 & 0 & 0 \\ 0 & 0.03^2 & 0 & 0 \\ 0 & 0 & 0.3^2 & 0 \\ 0 & 0 & 0 & 0.03^2 \end{bmatrix}.$$

It is planned to sample Q in a similar fashion as the sampling of R. Fig A shows the agreement for the ground truth for random walks with different step lengths and the chosen positional component of the process noise.

In addition to the Kalman filter, a Rauch-Tung-Striebel backwards smoothing filter is applied. Once the forward prediction-update step has been completed for a Kalman filter, we can use a Rauch–Tung–Striebel smoother to refine our earlier state estimates. This is a backwards step which starts from the final estimated state (i.e. the one which has been influenced by all observations) and works backwards creating optimal estimates of the hidden state, $\hat{X}_{t|T}$, and estimation error covariance, $P_{t|T}$. Note that these estimates have been computed given all observations.

The estimates are computed via the following recurrence relations:

$$\hat{X}_{t|T} = \hat{X}_{t|t} + L_t(\hat{X}_{t+1|T} - \hat{X}_{t+1|t}), \quad P_{t|T} = P_{t|t} + L_t(P_{t+1|T} - P_{t+1|t})L_t^T$$

where $L_t = P_{t|t}A^TP_{t+1|t}^{-1}$.

Biggles uses these estimates for the evaluation of the log-likelihoods on track observations.

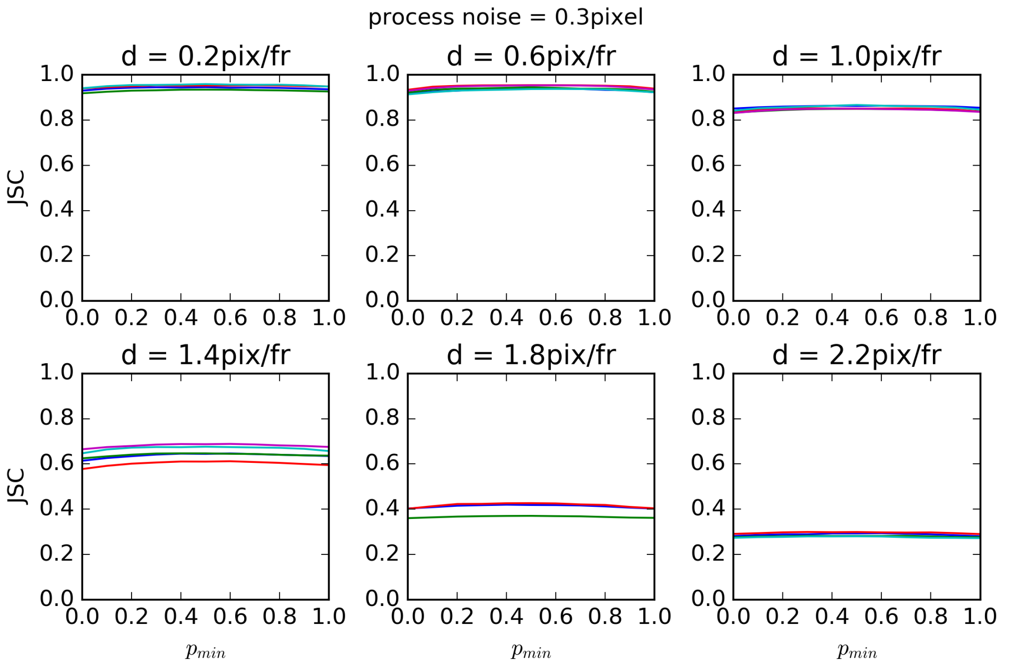

Fig A. Dependency on the process noise. A step length of $1 pixel/frame$ is equivalent to $D \approx 0.26 \mu m^2/s$ at a pixel size of $160nm$ and a frame rate of $20Hz$.

3.3 Track observations likelihood

The Kalman smoother outputs, for each candidate track, a set of maximally likely states. We assume that the evolution of states, beyond first order velocity, is due to the Gaussian noise process and so it is straightforward to write down the probability density for an individual observation, Y, corresponding to a predicted state, $\hat{X}$ by noting that $\hat{S}$, the innovation covariance or total error in the predicted observation, $\hat{Y} = B\hat{X}$, is given by $\hat{S} = B\hat{P}B^T + R$, see section 3.2. We therefore calculate the likelihood of Y assuming a Gaussian model:

$$P(Y|\hat{X}, \hat{P}, R) = \mathcal{N}(Y; B\hat{X}, \hat{S}).$$

The overall log probability density is therefore simply the sum of all the log probability densities for each observation associated with a track,

$$\ell(Y^i|k_i, \theta) = \sum_{j=1}^{N_o^i} \log P(Y_j^i|\hat{X}_{t(j)}, \hat{P}_{t(j)}, R), \tag{7}$$

where N_o^i is the number of observation of track k_i (i.e. the size of Y^i) and $t(j)$ is the time point of observation Y_j^i.

3.4 Clutter observations likelihood

The value of $\ell(Y^0|k_0, \theta)$ may be computed by assuming that each clutter observation arises independently and is uniformly likely over the field of view. Letting V be the total area (in square pixels) of the field of view, it follows that:

$$P(Y^0|k_0, \theta) = \prod_{t=1}^{T} \left(\frac{1}{V}\right)^{N_t^c}. \tag{8}$$

3.5 Parameter prior

The $\ell(\theta)$ term is computed as the sum of the (independent) parameter priors where:

- λ_b and λ_c have improper uninformative priors on them being positive.
- p_s and p_o have uniform priors over $(0, 1)$.
- R has an inverse Wishart prior $\mathcal{W}(\Phi, s)$ with parameters $\Phi = 2I$ and $s = 5$.

4 Sampling the parameters

Unlike track configuration, the parameters θ may be sampled directly since their posterior distribution is known and separable.

Sampling these parameters forms one half of the Gibbs sampler which implements the Biggles tracking algorithm; we sample a set of model parameters, θ, from the posterior distribution $P(\theta|\omega, Y)$ where ω is the partition and Y is the observed data.

The only parameter which depends directly on the observations is R, the remainder depend only on the track birth and death times and overall count of clutter observations. We may thus sample them from known conjugate parameter distributions for the distributions Specifically:

- $P(p_s|\omega) = f_\beta(p_s; 1 + N^s, 1 + N^d)$;
- $P(p_o|\omega) = f_\beta(p_o; 1 + N^o, 1 + N^s + N^b - N^o)$;
- $P(T\lambda_b|\omega) = f_\gamma(T\lambda_b; 1 + N^b, 1)$;
- $P(T\lambda_c|\omega) = f_\gamma(T\lambda_c; 1 + N^c, 1)$,

where T is the total frame count, $f_\beta(\cdot)$ is the PDF of the Beta distribution, $f_\gamma(\cdot)$ is the PDF of the Gamma distribution, $N^s = \sum_{t=1}^{T} N_t^s$ and similarly for N^d, N^o, N^b and N^c . Closed form solutions exist to sample from these distributions.

Sampling R is more difficult as it depends on the actual observations. To sample R , we first sample the underlying states of the dynamic model using the state estimates and covariance matrices generated by the Kalman smoother. This provides us with a state sample, X, drawn from $P(X|R, \omega, Y)$ with $\omega = \{k_0, \ldots, k_K\}$ where k_0 is the clutter. The usual distribution for sampling a covariance matrix is an inverse Wishart distribution, $P(R|X, \omega, Y) = \mathcal{W}^{-1}(R; \Phi', s')$ whose parameters may be calculated as:

$$\Phi' = \Phi + \sum_{i=1}^{K} \sum_{t \in T_i} (y_{i,t} - Bx_{i,t})(y_{i,t} - Bx_{i,t})^T, \quad s' = s + \sum_{i=1}^{K} l_k$$

where K is the number of tracks in the current partition, T_i is the set of time indices with an associated observation in track k_i, $y_{i,t}$ is the observation with time index t in track k_i, $x_{i,t}$ is the estimate of state with time index t in track k_i, B is the state observation matrix from the Kalman smoother and l_i are the number of observations in track k_i. The parameters of the prior are $s = 5$, $\Phi = 2I$.

5 Notes on partition distances, ergodicity and move design

Initially we note that whether the acceptance probability of the Metropolis-Hastings sampler is larger than zero for any two partitions ω' and ω and therefore if the sampling

chain can move from ω to ω' only depends on the proposal density $Q(\omega'|\omega)$ if . The target distribution for any partition is always larger than zero for most parameter values, i.e. $P(\omega|Y) = \int P(\omega, \theta|Y)d\theta > 0$ for all ω and data Y. This can be confirmed from equation (1).

5.1 Distances between partitions

In the following we address two similar questions; what is the maximum graph edit distance (GED) between any two partitions and what is the number of Biggles moves required to connect two partitions that are furthest away in terms of the Biggles moves. If we write $d(\omega, \omega')$ for the GED between ω, ω' and $b(\omega, \omega')$ for the minimum number of Biggles moves required to go from ω to ω', we are looking for

$$\max_{\omega,\omega' \in \Omega} d(\omega, \omega')$$

and

$$\max_{\omega,\omega' \in \Omega} b(\omega, \omega')$$

When applying the GED, we identify all tracks that have the same observations, i.e. we disregard eventual differences in the birth and death time as long as the observations are the same. The GED measures the distance between two partitions by counting the number of links in which the partitions differ. To transform one partition ω into another ω' can always be achieved by first deleting all links of ω, which results in the minimal partition ω^0, and then adding all links of ω'. The maximum possible GED in the set of all partitions, Ω, therefore may occur between two maximal partitions, ω and ω', that have no links in common. In the examples presented here, the maximal possible number of links in a partition is near but less than the number of observations N. That means the GED can be close to but will always be smaller than $2N$. Or more formally:

$$\max_{\omega,\omega' \in \Omega} d(\omega, \omega') \leq \max_{\omega \in \Omega} d(\omega^0, \omega) + \max_{\omega' \in \Omega} d(\omega^0, \omega') \leq N + N \leq 2N.$$

What is the maximum of the Biggles distance, $b(\omega, \omega')$? The shortest chain from the minimum partition ω^0 to any other partition has as many moves as the number of tracks in the target partition, since no move can change the number of tracks by more than one and the birth move can create any valid track. The maximum number of tracks occurs in such partitions where all tracks have at most 3 observations, since a track with 4 observation could be split into two, removing one link. Roughly speaking, the maximum possible number of tracks is near but always less than $N/2$ (depending on the data set), i.e. $b(\omega^0, \omega) < N/2$ for any ω. Since we can always move between any two partitions via the minimum partition, not more than N moves are needed to go from any partition to any other partition,

$$\max_{\omega,\omega' \in \Omega} b(\omega, \omega') < N$$

.

5.2 Distance based on Biggles moves

A proposal mass function Q could be constructed on the basis of graph edit operations. In our case we would get two moves that add or remove a single link, respectively. On the other hand any Q may imply a distance measure on Ω, if some weak assumptions are fulfilled. The Biggles proposal moves correspond to a distance measure on Ω, i.e.

$b(\omega, \omega')$, the minimum number of moves required to go from ω to ω', can be used as a distance function. We will see that the condition

$$Q(\omega|\omega') > 0 \iff Q(\omega'|\omega) > 0$$

is key for that. Biggles moves are defined via Q. If $Q(\omega'|\omega) > 0$ then it is possible that the Markov chain continues with the ω' given that the last sample was ω. Or, if the minimum number of Biggles moves required to go from ω to ω' is one, then it must be possible to directly draw ω' from Q given that the last sample is ω. We therefore have

$$b(\omega, \omega') = 1 \implies Q(\omega'|\omega) > 0 \quad .$$

However, in the current implementation of Biggles it is possible that the move construction does not lead to a new partition and the original partition is returned, hence

$$b(\omega, \omega') = 0 \implies Q(\omega'|\omega) > 0 \quad .$$

If the minimum number of Biggles moves that is required to go from one partition to another is larger than one, then the proposal density is zero otherwise the partitions in question could be connected with a single move:

$$b(\omega, \omega') > 1 \implies Q(\omega'|\omega) = 0 \quad .$$

Therefore

$$Q(\omega'|\omega) > 0 \iff b(\omega, \omega') \leq 1 \quad . \tag{9}$$

To be a distance function $b(\omega, \omega')$ needs to fulfil four conditions:

1. $b(\omega, \omega') \geq 0$ for all $\omega, \omega' \in \Omega$. This follows immediate from the definition of b (the minimum number of Biggles move to go from any partition ω to any other partition ω' is always zero or larger of course).

2. $b(\omega, \omega') = 0 \iff \omega = \omega'$. This follows immediately from the definition of b too (if the minimum number of Biggles moves to go from ω to ω' is zero, then ω is ω' and if ω is ω', then there are no moves required to go from ω to ω').

3. $b(\omega, \omega') = b(\omega', \omega)$ *(symmetry)*. Since Q is designed such that $Q(\omega|\omega') > 0 \iff Q(\omega'|\omega) > 0$, it follows with equation (9) that $b(\omega, \omega') \leq 1 \iff b(\omega', \omega) \leq 1$ and with condition 2 that $b(\omega, \omega') = 0 \iff b(\omega', \omega) = 0$. Therefore $b(\omega, \omega') = 1 \iff b(\omega', \omega) = 1$. If $(\omega_i)_{i=0}^k$ is a partition sequence from ω_0 to ω_k, with $b(\omega_i, \omega_{i+1}) = 1$ for $i = 0 \ldots k-1$, then the reverse sequence from ω_k to ω_0 fulfils $b(\omega_{i+1}, \omega_i) = 1$ for $i = 0 \ldots k-1$. If one sequence is a shortest of such sequences (there can be more than one shortest sequence), then its reverse sequence is a shortest sequence as well and we have $b(\omega_0, \omega_k) = b(\omega_k, \omega_0) = k$. Therefore, $b(\omega, \omega')$ is symmetric.

4. $b(\omega, \omega'') \leq b(\omega, \omega') + b(\omega', \omega'')$ *(subadditivity)*. Let $(\omega_i)_{i=0}^k$ with $\omega_0 = \omega$ and $\omega_k = \omega'$ be a shortest move sequence from ω to ω' and let $(\omega_i)_{i=k}^{k+l}$, with $\omega_{k+l} = \omega''$ be a shortest move sequence from ω' to ω''. Then $(\omega_i)_{i=0}^{k+l}$ is a (not necessarily shortest) move sequence from ω to ω'' of length $b(\omega, \omega') + b(\omega', \omega'') = k + l$. Therefore $b(\omega, \omega'')$, the length of a shortest move sequence from ω to ω'', is at most $b(\omega, \omega') + b(\omega', \omega'')$.

However, a distance measure based on the Biggles move is more complicated to implement and less general, therefore we didn't apply it. Biggles moves include all possibilities to add or remove a single link. Adding a single link can by accomplished by

the birth move, if both observations are clutter, by the extend move, if one observation is clutter and the other at the end of the track and the merge move, if both observations are part of a track. In other words, if ω and ω' only differ in 1 link, then it always holds $Q(\omega|\omega') > 0$. It always holds that

$$b(\omega, \omega') \leq d(\omega, \omega')$$

for all $\omega, \omega' \in \Omega$.

5.3 Ergodicity

We prove that the partition sampler is ergodic by following the argument by K. Murphy [1]. In order to show that the limiting distribution exists, we need to show that biggles chains are *irreducible* and *aperiodic*. A chain is irreducible if we can get from any partition to any other partitions, which we have shown above. A chain is aperiodic, if at least one partition in this chain is aperiodic, which in turn means there are two move sequences with length l_1 and l_2 that start and end in this partition so that the greatest common divisor of l_1 and l_2 is 1. Lets take a track with three observations. The following two sequences are always possible: death-birth with a length of 2 and reduce-death-birth with a length of 3. Therefore Biggles chains are aperiodic and the limiting distribution exists. Since Ω is finite and Biggles chains are irreducible, all ω are *recurrent* and *non-null*. Therefore Biggles chains are ergodic.

5.4 Number of modes of the target distribution and move design

Our first implementation of the algorithm did not include the transfer move and the cross-over move. To do a cross-over from ω to ω' without a special move requires two split moves and two merge moves, i.e. it requires to remove two links and to add two different links, creating intermediate partitions ω_1, ω_2 and ω_3. While it may be that $P(\omega|\theta, Y) \approx P(\omega'|\theta, Y)$, we may find $P(\omega_1|\theta, Y) \ll P(\omega|\theta, Y)$ and therefore the proposal ω_1 is likely to be rejected and it may take a large number of samples to reach ω'. The absence of the cross-over move creates a probability mass trench between ω and ω'. In other words, without the cross-over move the target distribution may have two modes that merge into one mode if the cross-over move is added. Hence, the modification of the proposal moves changes the number of local extrema of the target distribution. The value of target density of any partition remains unaffected, of course.

The design of the moves requires great care. For example during the execution of the birth move, rather than sampling the death time we could continuously sample observation until this fails, using a Poisson distribution to determine the time gaps between observations. However, the probability of sampling such a track would contain not just the probabilities of sampling the observations, but also the probability of failing to sample any observation at the end of the track. This fail probability is practically difficult to determine.

The design of the proposal mass function is critical for the performance of Biggles. While a short maximum distance between any two partitions is favourable, more important is the reduction of the number of modes and the reduction of $Q(\omega|\omega')$ for less acceptable proposals ω given ω'.

6 Technical Notes

6.1 Diffusion coefficient

We calculated the mean squared displacement $\langle r^2(\tau)\rangle$ for a partition ω as

$$\langle r^2(\tau)\rangle = \frac{1}{K}\sum_{i=1}^{K}\frac{1}{N_o^i-\tau}\sum_{j=1}^{N_o^i-\tau} d(Y_{j+\tau}^i, Y_j^i)^2 \quad .$$

As before, K is the number of tracks, N_o^i is the number of observations of track k_i and $d(Y_{j+\tau}^i, Y_j^i)$ is the Euclidean distance between observations $Y_{j+\tau}^i$ and Y_j^i. For readability, this equation assumes that there are no observation gaps in the tracks. The actual calculation skips expressions where the track has no pair of observations with time lag τ and the scaling factor $N_o^i - \tau$ is reduced accordingly. The diffusion coefficient D was estimated from

$$\langle r^2(\tau)\rangle = 4D\tau\,, \quad \tau \in \{1, 2\}.$$

The estimation uses the slope yielded by linear regression.

References

1. Kevin P. Murphy. 2012. Machine Learning. A probabilistic Perspective. MIT Press.
